# CircBRWD3 promotes non-small cell lung cancer progression through enhancing the stability of TRIM25 to increase p53 ubiquitin degradation and upregulating SPTBN2

**DOI:** 10.1038/s41419-026-09103-3

**Published:** 2026-07-16

**Authors:** Yanhua Zhu, Yuefeng He, Mei Xiao, Lei Guo, Xiao Liu, Xi Chen, Jiezhen Liu, Xu Chen, Yanliang Zhang

**Affiliations:** 1 Department of Clinical Laboratory, The First Affiliated Hospital of Kunming Medical University, Kunming, China; 2 Yunnan Key Laboratory of Laboratory Medicine, Kunming, China; 3 Yunnan Province Clinical Research Center for Laboratory Medicine, Kunming, China; 4 Department of Clinical Laboratory, The Third Affiliated Hospital of Kunming Medical University, Yunnan Cancer Hospital, Peking University Cancer Hospital Yunnan, Kunming, China; 5 School of Public Health, Kunming Medical University, Kunming, China

**Keywords:** Diagnostic markers, Non-small-cell lung cancer

## Abstract

Circular RNAs (circRNAs) play crucial roles in cancer progression and metastasis. However, the expression profiles and biological roles of circRNAs in non-small cell lung cancer (NSCLC) remain unclear. In this study, we found a novel circRNA, hsa_circ_0001936(circBRWD3), in NSCLC and investigated the biological role of circBRWD3 in NSCLC progression in vitro and in vivo. qRT-PCR was used to quantify the expression of circBRWD3 in NSCLC tissues, serum and cells. Cell growth curves, colony formation, Edu assay and transwell assays were conducted to study the function of circBRWD3. Finally, the molecular mechanism of circBRWD3 was revealed by Bioinformatic analyses, RNA pull-down analysis, RNA Fluorescence in situ hybridization (FISH), RNA-Binding Protein Immunoprecipitation (RIP), western blot, dual-luciferase reporter gene assays and rescue experiments. Our results showed that circBRWD3 was upregulated in NSCLC tumor tissues, patient serum and cell lines. CircBRWD3 knockdown inhibited NSCLC cells’ growth and metastasis both in vitro and in vivo, while circBRWD3 overexpression had the opposite effect. Mechanistically, circBRWD3 binds to the TRIM25 protein to enhance its stability and mediate ubiquitin degradation of the p53 protein. In addition, circBRWD3 acted as a sponge for miR-135a-5p and miR-135b-5p to regulate the expression of SPTBN2, which promoted NSCLC progression. Our findings uncovered a tumor oncogenic function of circBRWD3 in NSCLC through the regulation of TRIM25 protein stability and miR-135a-5p/miR-135b-5p/SPTBN2 axis. CircBRWD3 may act as a promising oncogenic biomarker and therapeutic target for NSCLC.

## Introduction

Lung cancer is the leading cause of cancer-related mortality worldwide, with a 5-year survival rate averaging only 25% [1]. Non-small cell lung cancer (NSCLC), which comprises approximately 85% of all lung cancer cases, is further classified into adenocarcinoma, squamous cell carcinoma, and large cell carcinoma [2]. Despite significant advancements in the diagnosis and treatment of NSCLC over the past decade, the prognosis remains poor, especially for metastatic cases, due to low early detection rates and limited therapeutic efficacy [3]. Understanding the molecular mechanisms underlying NSCLC progression and identifying novel biomarkers and therapeutic targets are essential for improving patient outcomes and developing more effective treatment strategies for NSCLC.

Circular RNAs (circRNAs) are non-coding RNAs with covalently closed-loop structures formed through pre-mRNA back-splicing, making them resistant to RNA exonucleases and more stable than linear RNAs, and highlighting their potential as reliable cancer biomarkers [4, 5]. Initially regarded as splicing byproducts, circRNAs are now recognized as key regulators of gene expression, exerting diverse biological functions such as sponging microRNAs (miRNAs), interacting with RNA-binding proteins (RBPs), modulating transcription, and even encoding peptides [6, 7]. In cancer, including NSCLC, circRNAs exhibit aberrant expression patterns and play critical roles in tumorigenesis, progression, and therapy resistance [8]. Certain circRNAs act as oncogenes by promoting cell proliferation, metastasis, and treatment resistance, while others serve as tumor suppressors by inhibiting these processes. For instance, circHERC1 was significantly upregulated in lung cancer tissues and cells and strikingly facilitated the proliferation, invasion and metastasis, while inhibiting the apoptosis of lung cancer cells [9]. Circ_POLA2 was highly expressed in lung cancer tissues and predicted a poor prognosis in lung cancer patients, functioning as a ceRNA by sponging miR-326 to promote lung cancer cell stemness and progression [10]. Conversely, circNDUFB2 was downregulated in NSCLC tissues and negatively correlated with NSCLC malignant features [11]. Elevated circNDUFB2 inhibits the growth and metastasis of NSCLC cells. Circ6834 was downregulated in NSCLC tumor tissues and cell lines, suppressed NSCLC progression by destabilizing ANHAK and regulating miR-873-5p/TXNIP axis [4]. These findings highlight the dual roles of circRNAs as oncogenes or tumor suppressors in NSCLC and underscore their potential as diagnostic biomarkers and therapeutic targets.

Increasing studies suggested that circRNAs participated in cancer biology via multiple mechanisms. For example, circVAPA acted as a sponge for miR-377-3p and miR-494-3p, modulating the insulin-like growth factor 1 receptor (IGF1R) and activating the phosphoinositide 3-kinase (PI3K)/protein kinase B (AKT) signaling pathway to accelerate NSCLC progression [12]. CircGUCY1A2 functions as a competitive miR-200c-3p sponge, enhancing PTEN expression and subsequently inhibiting the PI3K/AKT signaling pathway, thereby suppressing NSCLC progression [13]. Beyond serving as miRNA sponges, circRNAs can bind to RBPs, influence RNA splicing and gene transcription, function as scaffold molecules, and even translate into peptides [14]. For instance, circEIF4G3 bound to δ-catenin protein to promote its TRIM25-mediated ubiquitin degradation to inhibit β-catenin signaling to suppress gastric cancer progression [15]. CircCAPRIN1 directly bound signal transducer and activator of transcription 2 (STAT2) to activate Acetyl-CoA carboxylase 1 (ACC1) transcription, thus regulating lipid metabolism and facilitating colorectal cancer tumorigenesis [16]. Moreover, Liu et al. revealed that N6-methyladenosine-modified circIGF2BP3 promotes tumor immune evasion in NSCLC by suppressing CD8+ T-cell responses and facilitating PD-L1 deubiquitination [17]. Further investigation is warranted to elucidate their diverse roles in NSCLC progression and uncover the molecular mechanisms underlying their functions.

In this study, we have identified that the expression of circBRWD3 was increased in both tumor tissues and the serum of NSCLC patients. We investigated a correlation between circBRWD3 and tumor size. Additionally, we found the impact of circBRWD3 on the proliferation, migration, and invasion of NSCLC cells through experiments conducted in vitro and in vivo. We found that circBRWD3 exerted oncogenic roles in NSCLC progression by using gain- and loss-of-function studies. The results indicated that circBRWD3 functioned through the ceRNA pathway, as supported by rescue assays. These findings uncovered that circBRWD3 may be a potential biomarker and therapeutic target for human NSCLC.

## Materials and methods

### Patients and clinical specimens

A total of 29 paired tumor and adjacent non-tumor tissues from NSCLC patients, 104 serum samples from NSCLC patients, 100 serum samples from patients with benign lung disease and 108 serum samples from healthy donors were obtained from First Affiliated Hospital of Kunming Medical University between November 2022 and November 2023. None of the enrolled patients received chemotherapy, radiotherapy, immunotherapy, or other adjuvant therapy before surgery. Specimens were collected in accordance with institutional protocols. To minimize potential bias and batch effects during experimental procedures, all clinical specimens were processed and analyzed in a randomized and blinded manner. The study was conducted in accordance with the Declaration of Helsinki and its subsequent amendments. All methods were performed in accordance with the relevant guidelines and regulations. Written informed consent was obtained from all the participants, and the study was approved by the Institutional Ethical Committee of the First Affiliated Hospital of Kunming Medical University (Approval No. 2022-L-222).

### Cell culture

Human NSCLC lines (A549, H1299, H838, SPC-A1, and H1975), bronchial epithelial cells (HBE), and Human Embryonic Kidney 293 (HEK293T) cells were purchased from the Kunming Institute of Zoology (Kunming, China). A549, H1299, H838, SPC-A1, and H1975 cells were maintained in RPMI-1640 medium (Gibco) supplemented 10% fetal bovine serum (FBS) (Biological Industries, Israel). 16HBE cells were maintained in MEM medium (Gibco) with 10% FBS. All cells were cultured at 37 °C in a humidified 5% CO_2_ atmosphere.

### RNA fluorescence in situ hybridization (FISH)

An RNA FISH kit (GenePharma) was utilized following the manufacturer’s protocol, with the Cy3-labeled circBRWD3 RNA probe specifically designed and synthesized by GenePharma. Super-resolution microscopy was employed for cellular analysis, and the RNA probe sequence was listed in Table 1.

**Table 1:** The sequences of siRNAs and the probe.

Name	Target sequence (5′-3′)
si-NC	Sense: UUCUCCGAACGUGUCACGUTT
	Antisense: ACGUGACACGUUCGGAGAATT
si-circBRWD3-1	Sense: AGUUCUUCCAGGUGAUGGUTT
	Antisense: ACCAUCACCUGGAAGAACUTT
si-circBRWD3-2	Sense: UCUUCCAGGUGAUGGUGAGTT
	Antisense: CUCACCAUCACCUGGAAGATT
si-SPTBN2-1	Sense: CCCUGCAGAAAGAGAUUCATT
	Antisense: UGAAUCUCUUUCUGCAGGGTT
si-SPTBN2-2	Sense: GCUGCAGGAAAUGUGGAAATT
	Antisense: UUUCCACAUUUCCUGCAGCTT
si-TRIM25-1	Sense: GGAACAGUUAGUGGAUUUATT
	Antisense: UAAAUCCACUAACUGUUCCTT
si-TRIM25-2	Sense: GGAGCAGCUACAACAAGAATT
	Antisense: UUCUUGUUGUAGCUGCUCCTT
hsa_circ_0001936 FISH probe	CATCACCTGGAAGAACT+TTCACT+TTCCTCACCA+TCACCTGGAAGAACT+TTTACTACC+TCACCATCACC+TGGAAGA
hsa_circ_0001936 long probe	GTGATGGTGAGGTAGTAGAACAGGTAATTGGGCAGCAAACC AATGACCAAGATGAGAGCATTCTTGATGGAATAATCAGG GAGCTGCAGAGAGAACAAGACCTGAGACTAATTAATGAAG GAGATGTTCCACATTTACCAGTTAATAGAGCATACTCTGT TAATGGTGCTCTGAGAAGTCCAAACATGGACATATCTTCT TCCCCAAACATCAGGCTTCGAAGACATAGTAGTCAAATTG AAGGTGTTAGACAAATGCATAACAATGCTCCTCGGAGCCA GATGGCCACTGAAAGAGATCTCATGGCGTGGAGCAGAAGA GTGGTGGTCAATGAACTAAATAATGGGGTTAGTAGGGTAC AGGAAGAATGTCGAACTGCAAAAGGTGACATAGAAATCAG TCTTTATACAGTTGAAAAGAAGAAAAAGCCATCTTACACT ACTCAAAGGAATGATTATGAGCCTAGCTGTGGGCGTTCTT TACGCAGGACACAGCGCAAACGTCAGCATACTTACCAAAC ACGGTCCAACATAGAGCATAATTCACAGGCATCATGTCAA AATTCAGGTGTACAGGAAGACTCAGACAGTTCTTCAGAGG AAGATGAAACTGTTGGCACAAGTGACGCTTCGGTAGAGGA TCCTGTTGTTGAATGGCAAAGTGAAAGTTCTTCCAGGTGA TGGTGAGGTAGTAGAACAGGTAATTGGGCAGCAAAC
hsa_circ_0001936 antisense probe	GTTTGCTGCCCAATTACCTGTTCTACTACCTCACCATCAC CTGGAAGAACTTTCACTTTGCCATTCAACAACAGGATCCT CTACCGAAGCGTCACTTGTGCCAACAGTTTCATCTTCCTC TGAAGAACTGTCTGAGTCTTCCTGTACACCTGAATTTTGA CATGATGCCTGTGAATTATGCTCTATGTTGGACCGTGTTT GGTAAGTATGCTGACGTTTGCGCTGTGTCCTGCGTAAAGA ACGCCCACAGCTAGGCTCATAATCATTCCTTTGAGTAGTG TAAGATGGCTTTTTCTTCTTTTCAACTGTATAAAGACTGA TTTCTATGTCACCTTTTGCAGTTCGACATTCTTCCTGTAC CCTACTAACCCCATTATTTAGTTCATTGACCACCACTCTT CTGCTCCACGCCATGAGATCTCTTTCAGTGGCCATCTGGC TCCGAGGAGCATTGTTATGCATTTGTCTAACACCTTCAAT TTGACTACTATGTCTTCGAAGCCTGATGTTTGGGGAAGAA GATATGTCCATGTTTGGACTTCTCAGAGCACCATTAACAG AGTATGCTCTATTAACTGGTAAATGTGGAACATCTCCTTC ATTAATTAGTCTCAGGTCTTGTTCTCTCTGCAGCTCCCTG ATTATTCCATCAAGAATGCTCTCATCTTGGTCATTGGTTT GCTGCCCAATTACCTGTTCTACTACCTCACCATCAC

### Small interfering RNA (siRNA) transfection

Specifically targeted siRNAs were designed and synthesized by GnenPharma (Shanghai, China). Cells were seeded in 6-well plates at a density of 8 × 10^4^ cells per well and incubated for 18 h. Subsequently, siRNAs were transfected into cells using the RFect siRNA transfection reagent (Biogenerating Biotechnologies, Changzhou, China) under serum-deprived conditions. Cells were switched to complete medium 12 h after transfection and cultured for an additional 60 h. The cells were categorized into the negative control (NC), si-circBRWD3-1, and si-circBRWD3-2 groups. The sequences of siRNA were summarized in Table 1.

### RNase R treatment

To assess the circular characteristics and stability of circRNAs, total RNA (2 μg) was treated with 3 U/μg RNase R (Epicenter Technologies, Madison, WI, USA) at 37 °C for 30 min. For inactivation of RNase R, the reaction mixture was incubated at 65 °C for 20 min. Following enzymatic digestion, RNA was purified using the RNeasy MinElute Cleaning Kit (Qiagen, Germantown, MD, USA) to remove residual contaminants. Subsequently, the RNA was analyzed by reverse transcription-quantitative PCR (RT-qPCR) to evaluate the abundance of circBRWD3 and β-actin mRNAs. The sequences of the primers can be found in Table 2.

**Table 2:** Primer sequences for qRT-PCR.

Name	Primer sequence (5′-3′)
CircBRWD3	F: 5′-CTTACCAAACACGGTCCAACATAG-3′
	R: 5′-TTCTACTACCTCACCATCACCTGG-3′
β-actin	F: 5′-GCCGAGGACTTTGATTGCAC-3′
	R: 5′-TGGACTTGGGAGAGGACTGG-3′
RPLPO	F: 5′-GGTCATCCAGCAGGTGTTCG-3′
	R: 5′-GCAGACAGACACTGGCAACA-3′
SPTBN2	F: 5′-ACATCCACGCCGACAAGATT-3′
	R: 5′-GCGCTGCGTCTTGATTCTTC-3′
Cyclin D1	F: 5′-CAGATCATCCGCAAACACGC-3′
	R: 5′-AAGTTGTTGGGGCTCCTCAG-3′
Cyclin D3	F: 5′-TACCCGCCATCCATGATCG-3′
	R: 5′-AGGCAGTCCACTTCAGTGC-3′
Cyclin E2	F: 5′-GATGGTGCTTGCAGTGAAGAGGA-3′
	R: 5′-ACAAGGCAGCAGCAGTCAGTATT-3′
CDK4	F: 5′-CTGGACACTGAGAGGGCAAT-3′
	R: 5′-TGGGAAGGAGAAGGAGAAGC-3′
Vimentin	F: 5′-AACCTGGCCGAGGACATCA-3′
	R: 5′-TCAAGGTCAAGACGTGCCAGA-3′
Slug	F: 5′-CTGGTTGCTTCAAGGACACATTAG-3′
	R: 5′-CAGCCAGATTCCTCATGTTTGTG-3′
Twist	F: 5′-GTACATCGACTTCCTCTACCAG-3′
	R: 5′-CATCCTCCAGACCGAGAAG-3′
miR-135a-5p	F: 5′-GCTATGGCTTTTTATTCCTATGTGA-3′
miR-135b-5p	F: 5′-GCTATGGCTTTTCATTCCTATGTGA-3′
U6	F: 5′-CTCGCTTCGGCAGCACA-3′
TRIM25	F: 5′-GTCTCTACCCAGAACAGTTTCC-3′
	R: 5′-ATCCAACACAGGCTGATTCC-3′

### RNA pull-down assay

Following the manufacturer’s instructions of the RNA pull-down kit (Bersinbio, China), 1 × 10⁷ cells were harvested and lysed in lysis buffer. Then, the streptavidin-coated magnetic beads (Bersinbio, China) were incubated with the biotin-labeled circBRWD3 or the full-length antisense strand probes, which served as the control probe at room temperature. We incubated the cell lysates with RNA probe-coupled beads and collected the proteins pulled down using the elution buffer. Eventually, the retrieved proteins were further identified or validated by silver staining or Western blot.

### RNA immunoprecipitation (RIP) assay

RIP assay was performed using magnetic beads (MCE, NJ, USA). Approximately 1 × 10^7^ cells were collected and lysed with RIP lysis buffer. Cell lysates were then incubated with the anti-Ago2 and TRIM25 antibody at 4 °C overnight. Magnetic beads were pre-incubated with the mixture of cell lysates and antibody for 6 h at 4 °C. The immunoprecipitated RNA complexes were purified and quantified by quantitative real-time PCR (qRT-PCR). Normal rabbit IgG was used as the negative control.

### Dual luciferase reporter assay

HEK293T cells were cultured in 24-well plates and transfected with miRNA-binding site containing wild-type (WT) or mutant (MUT) vector, and predicted miRNA mimics or negative controls (NC) (GenePharma, Shanghai, China). After 24 h, the luciferase activity was measured using the dual-luciferase reporter assay system (Beyotime, Shanghai, China). Firefly luciferase intensity was normalized to Renilla luciferase, and fold changes relative to the NC were calculated.

### Cell colony formation assay

Lung cells were seeded into 6-well plates at a concentration of 1000 cells per well and maintained for 48 h. Subsequently, circBRWD3 siRNA was transfected into cells for 12 h, and then the complete medium was replaced. On the 10th day, cell colonies were fully fixed with 4% paraformaldehyde and stained with crystal violet. The number of colonies was counted.

### EdU assays

EdU assays were performed to evaluate the cells’ proliferation ability based on the manufacturer’s instructions regarding the use of the Cell Light EdU 555 Kit (Beyotime, Beijing, China). Briefly, transfected cells were seeded into 12-well plates at a density of 1 × 10^4^ cells/well. After adding EdU, the cells were cultured for 2 h, fixed with 4% PFA and stained with the kit following the manufacturer’s instructions. Images were captured using an Olympus microscope (IX53, Olympus, Tokyo, Japan).

### Cell migration and invasion assay

Cell migration and invasion were assessed using 24-well transwell chambers with 8-μm pore polycarbonate membranes (Corning, NY, USA) with or without Matrigel (Corning, NY, USA). 4 × 10⁴ transfected cells (in 200 μL serum-free medium) for migration and 8 × 10^4^ transfected cells were seeded into the upper chamber, while 600 μL of medium with 15% FBS was added to the lower chamber. After incubation for 24 h, cells that migrated or invaded were fixed with 4% paraformaldehyde, stained with 0.1–0.25% crystal violet, and non-migrated or non-invaded cells on the upper membrane surface were removed. Migrative or invasive cells were photographed, imaged and counted under an Olympus microscope.

### Western blot analysis

Lung cells transfected for 72 h were collected and lysed using RIPA buffer enriched with 1% protease inhibitors and phosphatase inhibitors. Equivalent quantities of proteins were separated on 10% SDS-polyacrylamide gels and transferred onto polyvinylidene fluoride membranes (Roche Diagnostics, Mannheim, Germany). The membranes were then blocked with protein-free rapid blocking solution (EpiZyme, PS108, China) for 20 min. Subsequently, the membranes were incubated overnight with primary antibodies at 4 °C, followed by a 2-h incubation with an HRP-conjugated secondary antibody at room temperature. BeyoECL Plus chromogenic substrate (Beyotime, Beijing, China) was used to visualize the bands. The source of antibodies can be found in Table 3.

**Table 3:** The source of antibodies.

Antibody name	Vendor and catalog number
p53 monoclonal antibody Phospho-p53 (Ser15) antibody Phospho-p53 (Ser392) antibody	Santa Cruz Biotechnology (USA), SC-126 Affinity Biosciences (China), AF3705 Huabio Biotechnology (China), ET1606-24
Anti-Argona 2 monoclonal antibody	Huabio Biotechnology (China), ET1702-39
Ubiquitin monoclonal antibody	Proteintech Group, Inc. (USA), 80992-1-RR
TRIM25 polyclonal antibody TRIM25 monoclonal antibody	Huabio Biotechnology (China), HA500104 Abcam Limited (UK), ab167154
SPTBN2 monoclonal antibody	Abcam Limited (UK), ab238055
β-actin monoclonal antibody Goat anti-Rabbit IgG Goat anti-Mouse IgG	Abclona (China), AC026 Huabio Biotechnology (China), HA1001 Huabio Biotechnology (China), HA1006

### In vivo animal studies

For the xenograft tumor model, 4-week-old male BALB/c nude mice were obtained from the specific pathogen-free (SPF) Biotechnology (Beijing, China) and housed in a SPF animal facility with strictly controlled conditions: temperature of 22 ± 2 °C, relative humidity of 50 ± 5%, and a 12 h light/12 h dark cycle. All mice were given free access to standard rodent chow and sterilized water. All animal procedures were performed in strict accordance with the National Institutes of Health guidelines for the care and use of laboratory animals. Mice were randomly assigned into two groups (*n* = 5 per group) and subcutaneously injected with 5 × 10^6^ H1299 cells per mouse, transfected with circBRWD3 shRNA or control vector. Tumor size was monitored at regular intervals and calculated using the formula: volume = 0.5 × length × width^2^. At the end of the experiment, tumor tissues were collected for hematoxylin and eosin (H&E) staining and immunohistochemistry (IHC) analysis. All animal protocols were reviewed and approved by the Animal Ethics Committee of Kunming Medical University (Approval No. kmmu20231493).

### Statistical analysis

All statistical analyses were conducted using SPSS software (version 22.0, Chicago, IL, USA) and GraphPad Prism (version 5.0 or 7.0, La Jolla, CA, USA). Continuous variables were expressed as mean ± standard deviation (SD) from at least three independent experiments, and differences between two groups were assessed using a two-tailed Student’s *t*-test, whereas comparisons among multiple groups were conducted using one-way ANOVA followed by Tukey’s post-hoc test. Pearson’s *χ*² test was used to analyze categorical variables. The area under the ROC curve (AUC) was calculated to evaluate predictive performance. Correlations were analyzed using Pearson’s correlation coefficients. Statistical significance was defined as *p* < 0.05.

## Results

### The characteristic of circBRWD3 in NSCLC cells

**Fig. 1:**
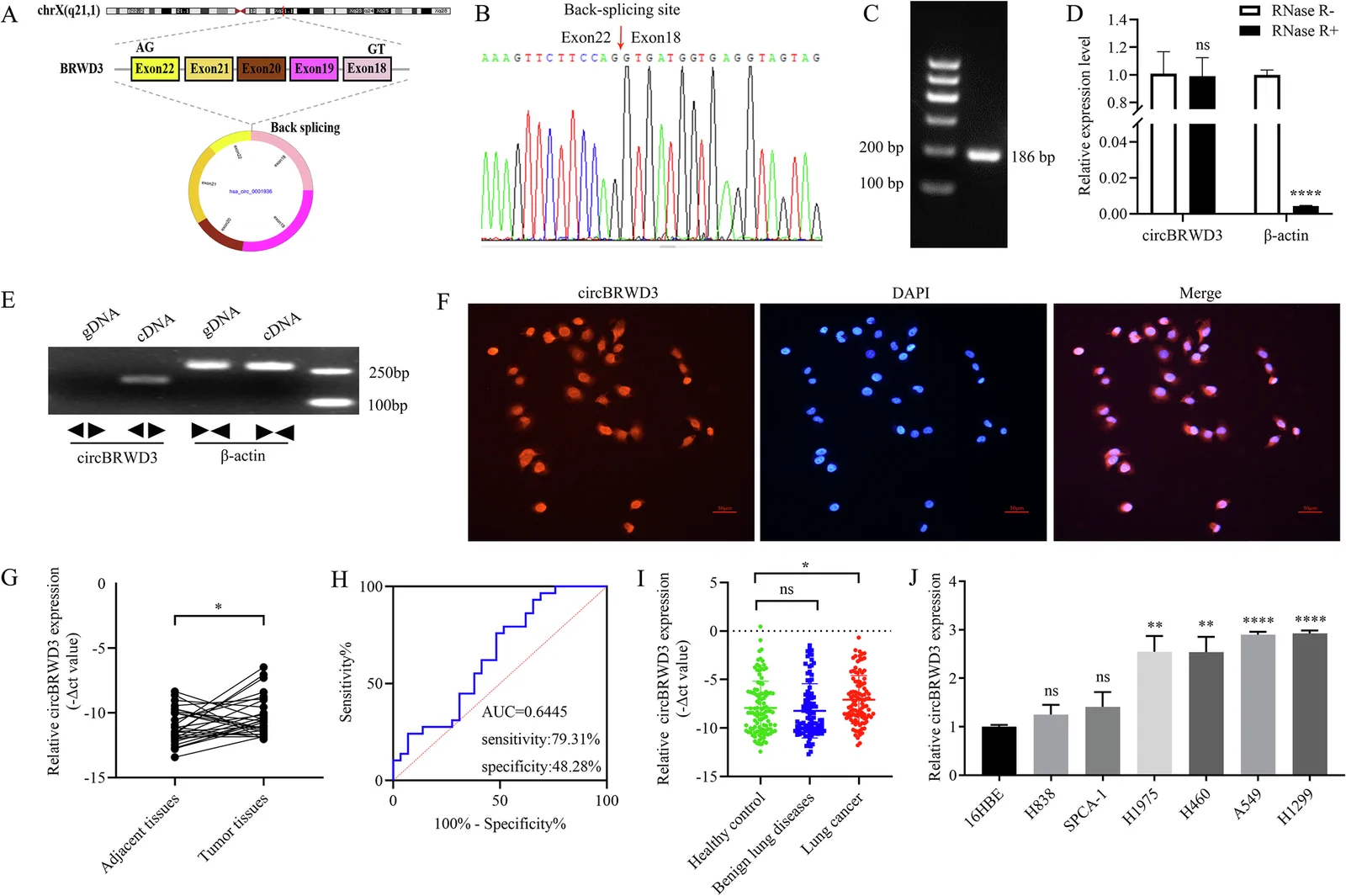
CircBRWD3 was upregulated in NSCLC. **A** Genomic location of circBRWD3. CircBRWD3 was formed by the back-splicing of exons 18–22 of BRWD3. **B** The back-splice junction site of circBRWD3 was identified by Sanger sequencing. **C** PCR of cDNA using divergent primers of circBRWD3. **D** The stability of circBRWD3 and β-actin mRNA was detected by RNase R degradation assay. Data are shown as means ± SD (*n* = 3). **E** PCR assay of gDNA and cDNA using divergent and convergent primers of circBRWD3. **F** Detection of the subcellular localization of circBRWD3 in NSCLC cells by FISH. **G** qRT-PCR analysis of circBRWD3 expression in 29 pairs of NSCLC tissues and adjacent non-tumor tissues. **H** ROC curve of circBRWD3 in distinguishing NSCLC and normal tissues. **I** qRT-PCR analysis of circBRWWD3 expression in the serum of NSCLC cancer patients, benign lung disease patients and healthy controls. **J** The expression level of circBRWD3 in NSCLC cells. *p < 0.05, **p < 0.01, * **p < 0.001, ns no significance.

To identify the deregulated circRNAs in NSCLC, we performed RNA chip analysis by using matched tumor tissues and adjacent non-tumor tissues from NSCLC patients. We focused on circBRWD3 because it has not been reported in NSCLC until now. CircBRWD3 was transcribed from the host gene BRWD3 by back-splicing of exon 18 to 22 with 667 nucleotides, which was validated by Sanger sequencing of the PCR product (Fig. 1A). Sequencing results confirmed the existence of back-splicing site in divergent primers-amplified PCR product (Fig. 1B). We designed convergent primers across the junction site and PCR analysis showed that circBRWD3 could be amplified by divergent primers in cDNA (Fig. 1C). The results of RNase R exonuclease treatment showed that circBRWD3 was resistant to their digestion (Fig. 1D). Moreover, the results of PCR with divergent primers showed that circBRWD3 was amplified from cDNA but not from the genomic DNA of NSCLC cells (Fig. 1E). Collectively, these datas supported that circBRWD3 was a circular RNA with high stability. To assess the subcellular distribution of circBRWD3 in NSCLC cells, we conducted RNA fluorescence in situ hybridization (FISH) experiments. The results showed that circBRWD3 was localized in both the cytoplasm and the nucleus of NSCLC cells (Fig. 1F).

### CircBRWD3 was upregulated in NSCLC tissues and serum

We further detected the expression of circBRWD3 in paired tumor tissues and adjacent non-tumor tissues from NSCLC patients (*n* = 29). qRT-PCR results showed that circBRWD3 expression levels were increased in tumor tissues compared to in adjacent non-tumor tissues (Fig. 1G). We then evaluated the clinical significance of circBRWD3 in NSCLC and found that high levels of circBRWD3 were related to tumor size in NSCLC patients (Table 4). The area under the receiver operating characteristic curve (AUC) was 0.6445 between NSCLC patients and healthy individuals, with a sensitivity of 79.31% and a specificity of 48.28% (Fig. 1H). Meanwhile, we observed that the expression of circBRWD3 was significantly upregulated in the serum of NSCLC patients compared to those of benign lung disease patients and healthy donors (Fig. 1I). Furthermore, qRT-PCR was performed to detect the expression of circBRWD3 in the tissues of NSCLC patients and NSCLC cell lines. We found that circBRWD3 expression was upregulated in NSCLC cell lines (SPC-A1, A549, H1975, H1299, and H838) compared to and 16HBE (Fig. 1J). In summary, these data indicate that circBRWD3 may be used as a biomarker for early diagnosis of NSCLC.

**Table 4:** The relationship between the expression level of circBRWD3 in lung cancer tissues (−ΔCt) and the clinical and pathological characteristics of lung cancer patients.

Features	Number	High	Low	*p* value
Gender				
Male	12	8	4	1.000
Female	17	11	6	
Age (years)				
<60	17	12	5	0.694
≥60	12	7	5	
Tumor size (cm)				
<3	6	1	5	0.011*
≥3	23	18	5	
Lymphatic metastasis				
N0	14	10	4	0.700
N1–N3	15	9	6	
Invasion depth				
T1 and T2	5	2	3	0.306
T3 and T4	24	17	7	
TNM stage				
Ⅰ and Ⅱ	14	9	5	1.000
Ⅲ and Ⅳ	15	10	5	

*P < 0.05

### Silencing of circBRWD3 inhibited the proliferation and metastasis of NSCLC cell lines

**Fig. 2:**
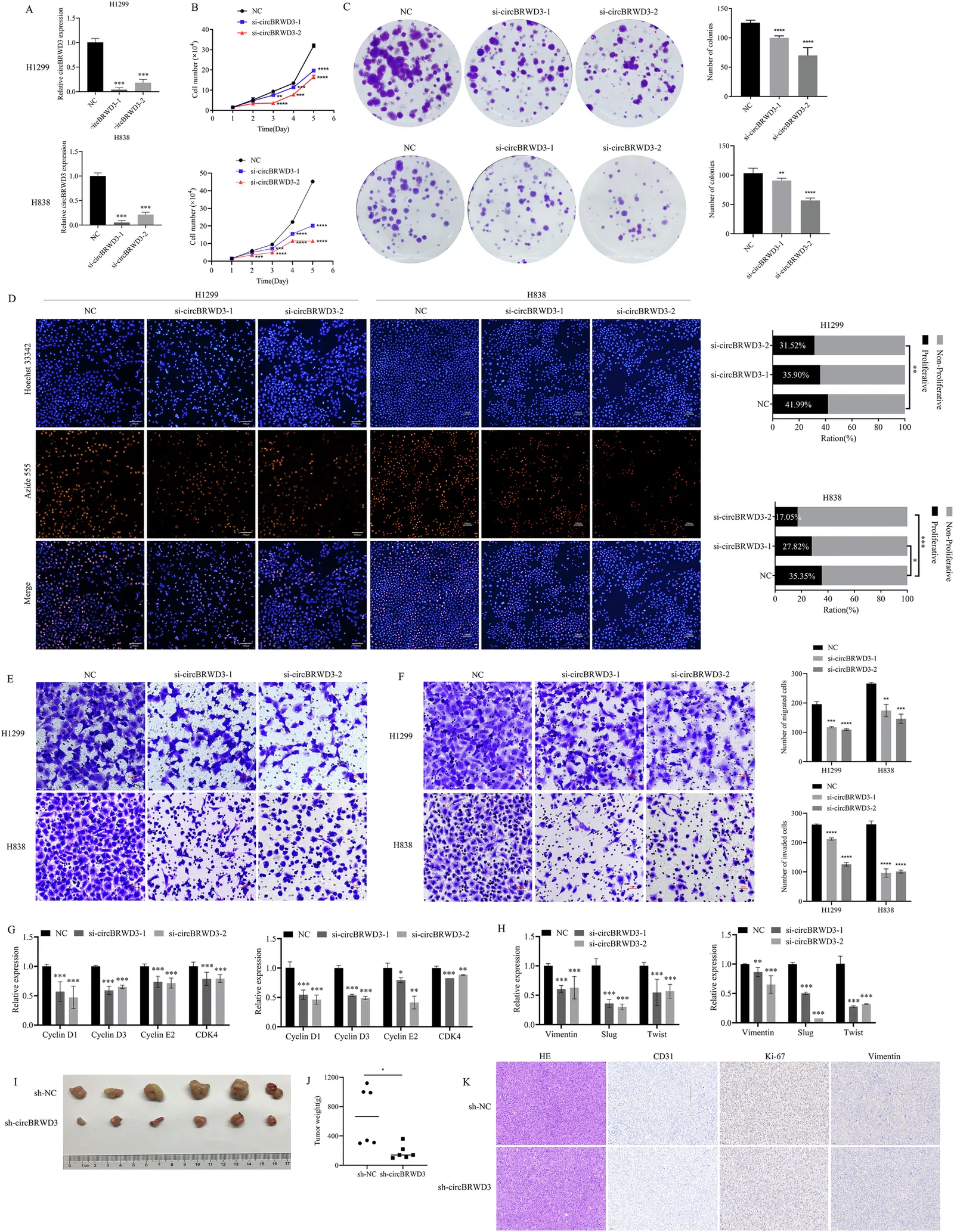
Silencing of circBRWD3 inhibited the proliferation and metastasis of NSCLC cell lines. **A** qRT-PCR analysis of circBRWD3 in NSCLC cells treated with two siRNAs against circBRWD3. **B** The cell growth curves. **C** The impact of circBRWD3 knockdown on NSCLC cell proliferation was determined by cell colony formation assay. **D** Edu assay. Transwell migration (**E**) and matrigel invasion assays (**F**) for transfecting with si-circBRWD3. **G**, **H** qRT-PCR of proliferation and EMT index after treatment with si-circBRWD3. **I**, **J** The tumor weight in mice of the control and circBRWD3 knockdown groups. **K** Sliced mouse tumor tissues were subjected to H&E, CD31, Ki67, and Vimentin staining (scale bars = 100 μm).

To explore the biological functions of circBRWD3 in NSCLC, we performed loss-of-function studies by using specific siRNAs that target the back-splice junction site of circBRWD3 and verified the knockdown efficiency in NSCLC cells by qRT-PCR (Fig. 2A). The results of cell growth curves, cell colony formation assays and Edu assays showed that circBRWD3 knockdown remarkably inhibited NSCLC cells’ proliferation (Fig. 2B–D). In addition, the results of Transwell assays showed that circBRWD3 knockdown inhibited NSCLC migration and invasion (Fig. 2E, F). Subsequently, the results of the qRT-PCR assay showed that circBRWD3 knockdown downregulated the expression of cyclin D1, cyclin D3, cyclin E2, and CDK4 in NSCLC cells (Fig. 2G), the expression of Vimentin, Slug, and Twist, as well as epithelial-mesenchymal transition (EMT) factors (Fig. 2H). We then established mouse subcutaneous xenograft tumor models to analyze the effect of circBRWD3 on NSCLC growth in vivo. The results showed that circBRWD3 knockdown could obviously inhibit both tumor size and weight compared to the negative controls (Fig. 2I, J). In addition, the decreased number of proliferating tumor cells and angiogenesis in mouse tumor tissues of the circBRWD3 knockdown group were validated by IHC staining for Ki67 and CD31 (Fig. 2K). Collectively, these results suggested that circBRWD3 exerted oncogenic roles in NSCLC progression.

### CircBRWD3 overexpression promoted the progression of NSCLC

**Fig. 3:**
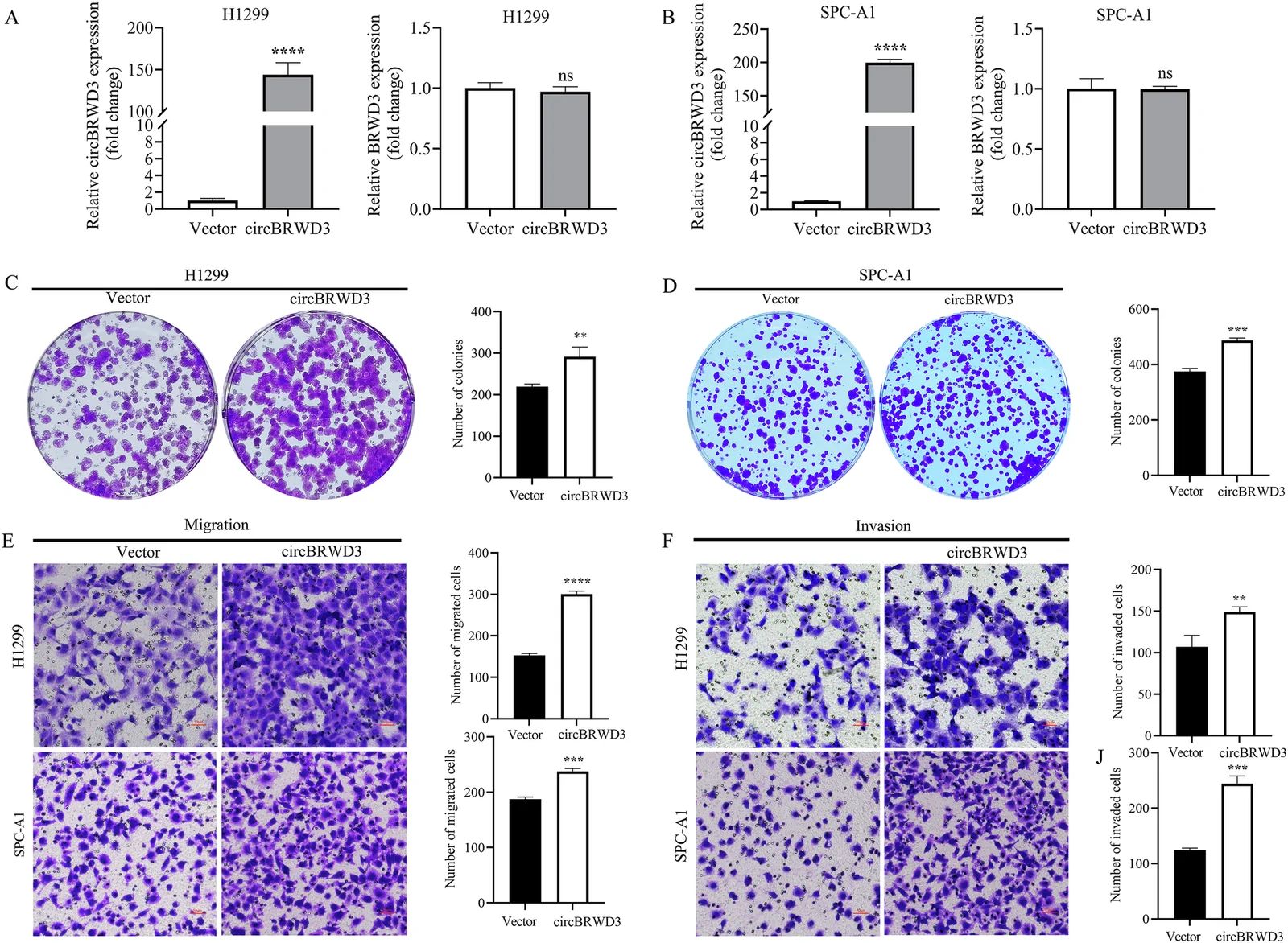
CircBRWD3 overexpression promoted the progression of NSCLC in vitro. **A**, **B** The efficiency of circBRWD3 after transfecting overexpressing circBRWD3. **C**, **D** Cell colony formation assay of GC cells transfected with vector or circBRWD3 overexpressing was performed to evaluate cell proliferative ability. **E**, **F** Transwell migration and matrigel invasion assays.

To further verify the effects of circBRWD3 on the malignant progression of NSCLC, we overexpressed circBRWD3 in NSCLC cells. The efficiency of circBRWD3 overexpression was verified by qRT-PCR (Fig. 3A, B). Conversely, results showed that overexpression of circBRWD3 markedly promoted the proliferation, migration, and invasion abilities of NSCLC cells (Fig. 3C–F). Collectively, these results suggested that circBRWD3 exerted oncogenic roles in NSCLC progression.

### CircBRWD3 interacted with TRIM25 protein

**Fig. 4:**
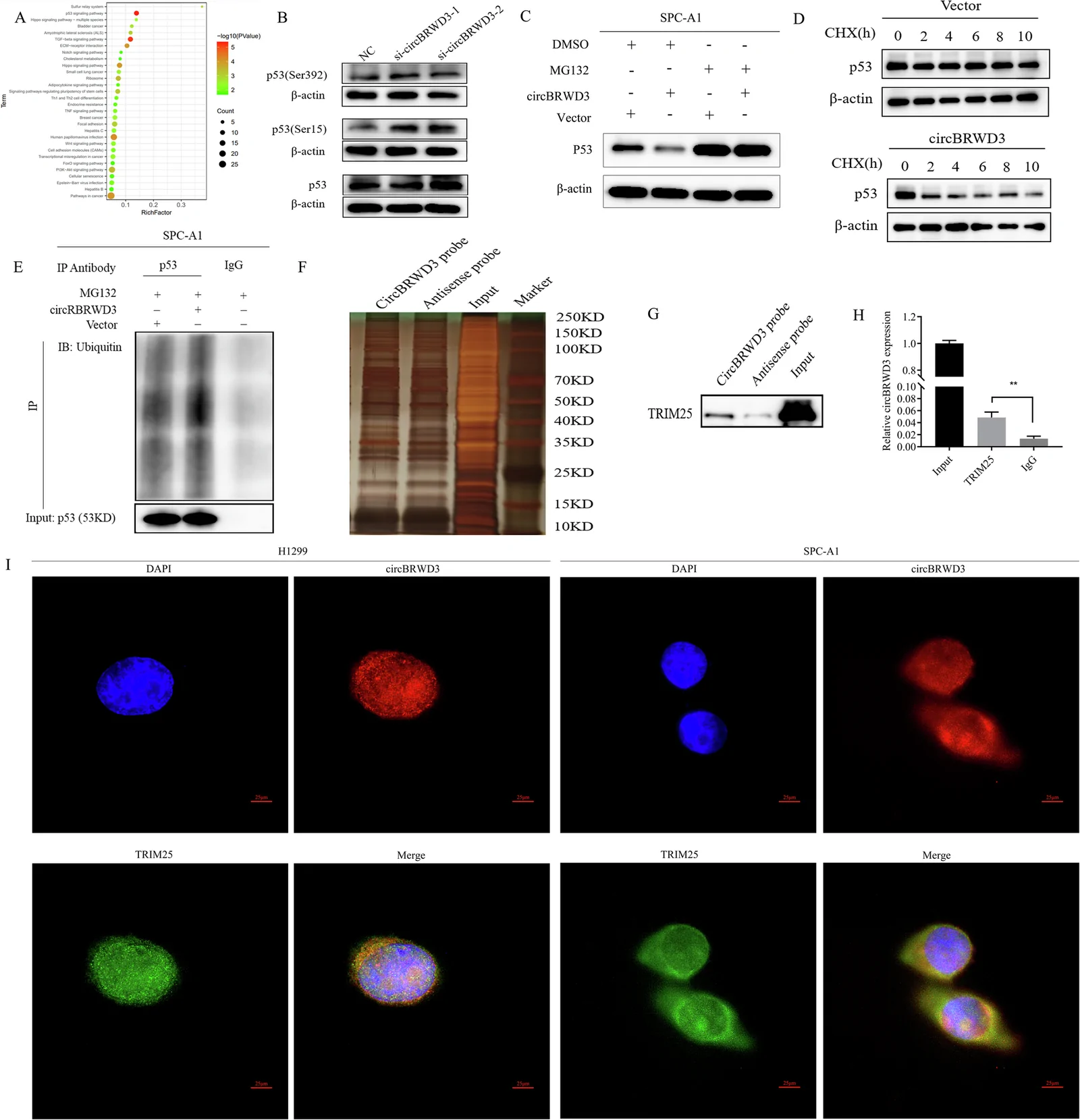
CircBRWD3 interacted with the TRIM25 protein. **A** GO enrichment analysis knockdown of circBRWD3 in lung cancer cells revealed the upregulated signaling pathways via RNA Sequence. **B** Western blot analysis was used to detect the expression levels of p53-related pathway proteins. **C** MG132 (20 µM, 6 h) treatment for p53 expression in SPC-A1 cells overexpressing circBRWD3. **D** CHX assay for determining the half-life of p53 protein in SPC-A1 cells overexpressing circBRWD3. **E** The levels of ubiquitinated p53 in SPC-A1 cells overexpressing circBRWD3. **F** RNA pull-down assay was performed in NSCLC cells using a circBRWD3 sense probe (circBRWD3 probe) and an antisense probe (ctrl probe). **G** RNA pull-down assay and western blotting validated the existence of TRIM25 in the precipitates pulled down with the circBRWD3 probe. **H** RIP assay validated the interaction between TRIM25 and circBRWD3. **I** RNA FISH and immunofluorescence analysis indicated the colocalization of circBRWD3 (red) and TRIM25 (green) in NSCLC cells.

Previous studies have suggested that circRNAs can function as miRNA sponges, interact with RNA-binding proteins, and serve as templates for protein translation [18, 19]. Based on these prior findings, we further explored the potential mechanism by which circBRWD3 functioned as an oncogene in NSCLC. To elucidate the functional role of circBRWD3 in NSCLC, RNA sequencing was performed to compare gene expression profiles between circBRWD3 knockdown (sh-circBRWD3) and control (sh-NC) lung cancer cells. Gene Ontology (GO) and Pathway enrichment analyses revealed that the p53 signaling pathway was significantly upregulated upon circBRWD3 silencing (Fig. 4A). Western blot validation further confirmed that knockdown of circBRWD3 increased the protein levels of total p53 and its phosphorylated forms at Ser392 and Ser15 (Fig. 4B), indicating that circBRWD3 depletion activated the p53 pathway.

Mechanistically, we investigated how circBRWD3 regulates p53. Treatment with the proteasome inhibitor MG132 abolished the circBRWD3 overexpression-induced downregulation of p53 (Fig. 4C), suggesting that circBRWD3 mediated the proteasome-dependent degradation of p53. Cycloheximide (CHX)-chase assays (30 μg/mL, 0–10 h) revealed that circBRWD3 overexpression accelerated p53 protein degradation, thereby shortening its half-life (Fig. 4D). Co-IP and ubiquitination assays further showed that circBRWD3 overexpression significantly enhanced p53 ubiquitination in SPC-A1 cells compared to the vector control (Fig. 4E). Collectively, these data indicated that circBRWD3 modulates p53 stability via ubiquitin-dependent degradation, which was critical for its regulation of NSCLC biological behaviors.

To evaluate whether circBRWD3 interacted with proteins to promote NSCLC progression, we conducted an RNA pull-down assay using a biotin-labeled sense or antisense probe of circBRWD3. The proteins enriched by the pull-down assay were separated by Sodium Dodecyl Sulfate PolyAcrylamide Gel Electrophoresis and visualized by silver staining (Fig. 4F). Western blot analysis following the RNA pull-down assay confirmed the direct interaction between circBRWD3 and TRIM25 in NSCLC cells (Fig. 4G). Importantly, RIP assay results confirmed that circBRWD3 was specifically enriched in the TRIM25-immuno-precipitated complex in NSCLC cells (Fig. 4H). Finally, RNA FISH and immunofluorescent staining results showed that circBRWD3 co-localized with TRIM25 in the nucleus of NSCLC cells (Fig. 4I). These results suggested that circBRWD3 directly interacted with TRIM25.

### CircBRWD3 enhanced the stability of TRIM25 to mediate ubiquitin degradation of p53

**Fig. 5:**
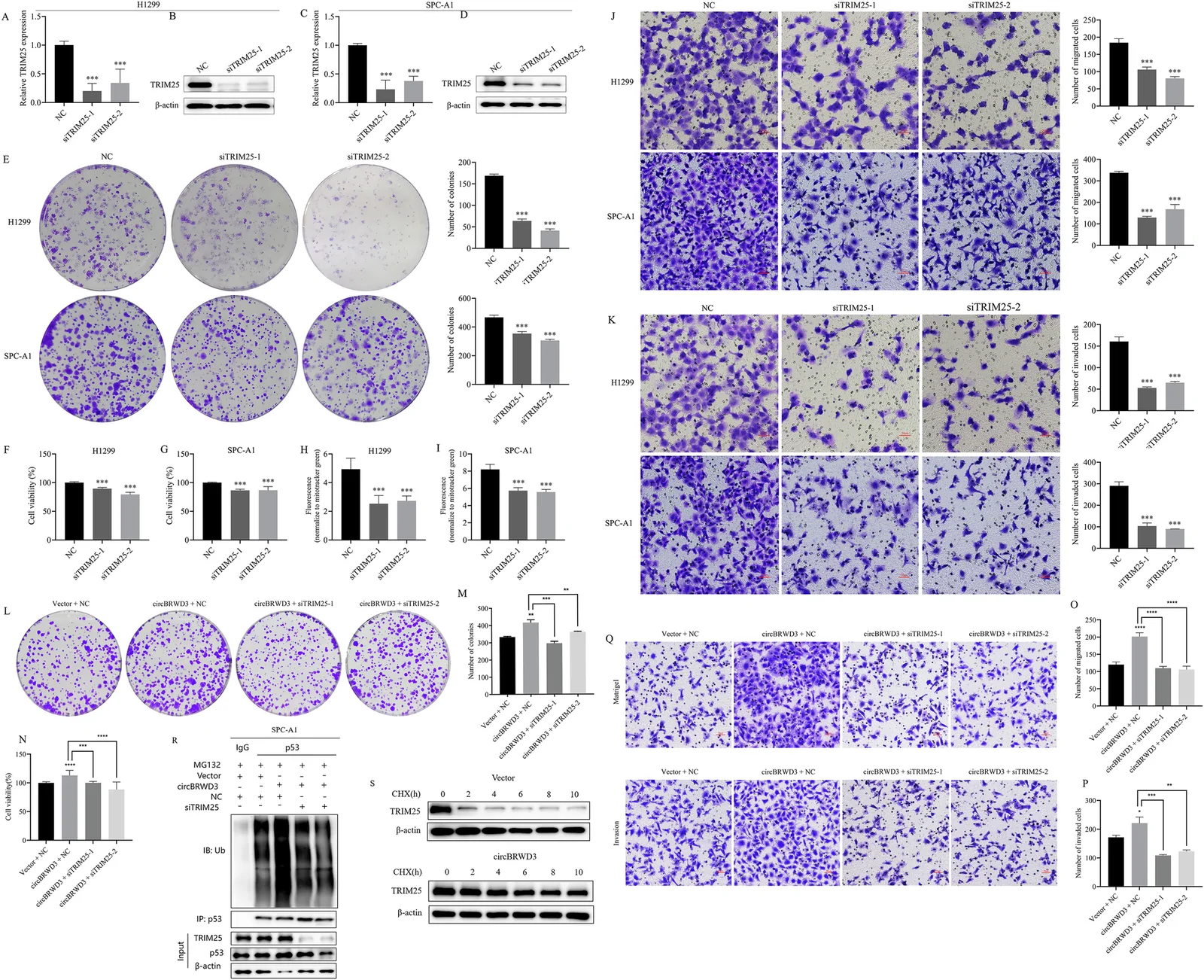
CircBRWD3 enhanced the stability of TRIM25 to mediate ubiquitin degradation of p53. **A**–**D** The expression of TRIM25 mRNA and protein was detected after TRIM25 knockdown in NSCLC cells. **E** Colony formation assay for NSCLC cells. **F**, **G** CCK-8 assay was performed to assess cell vitality in NSCLC cells. **H**, **I** Mitochondrial membrane potential assay was used to analyze the situation of cell apoptosis. **J**, **K** Cell migration and invasion assays of NSCLC cells treated with TRIM25 knockdown. Colony formation assays (**L**, **M**), CCK-8 assay (**N**), transwell migration and matrigel invasion assays (**O**–**Q**) were carried out for rescue experiments. **R** The ubiquitination of p53 in circBRWD3 overexpression SPC-A1 cells with TRIM25 knockdown. **S** Protein biosynthesis in SPC-A1 cells was blocked with cycloheximide (CHX). The protein levels of TRIM25 in SPC-A1 cells of the indicated groups were determined at the indicated time points by western blot.

Previous research works have indicated that TRIM25 is involved in tumor progression. TRIM family proteins, including TRIM25, promote the degradation of their substrates via the ubiquitin proteasome pathway and might serve as biomarkers and therapeutic targets for tumors [20, 21]. Further investigation of the biological function of TRIM25 in NSCLC cells through loss-of-function studies further showed that TRIM25 knockdown decreased NSCLC cell proliferation, viability, cellular mitochondrial membrane potential, migration, and invasion (Fig. 5A–K). To verify the regulatory effect of circBRWD3 on TRIM25, we performed a cell function compensation experiment by knocking down TRIM25 in SPC-A1 cells overexpressing circBRWD3. Further experiments revealed that the absence of TRIM25 could partially reverse the increase in cell proliferation, migration and invasion caused by circBRWD3 overexpression in SPC-A1 cells (Fig. 5L–Q). More importantly, we observed that TRIM25 knockdown significantly abrogated the circBRWD3 overexpression-induced increase in p53 ubiquitination levels in SPC-A1 cells (Fig. 5R). Meanwhile, CHX-chase assays showed that circBRWD3 stabilized the TRIM25 protein (Fig. 5S). These results showed that circBRWD3 promoted the ubiquitination and degradation of p53 by enhancing the stability of TRIM25. Collectively, circBRWD3 promoted NSCLC progression by stabilizing TRIM25, thereby enhancing TRIM25-dependent p53 ubiquitination and degradation.

### CircBRWD3 acted as a sponge for miR-135a-5p/miR-135b-5p

**Fig. 6:**
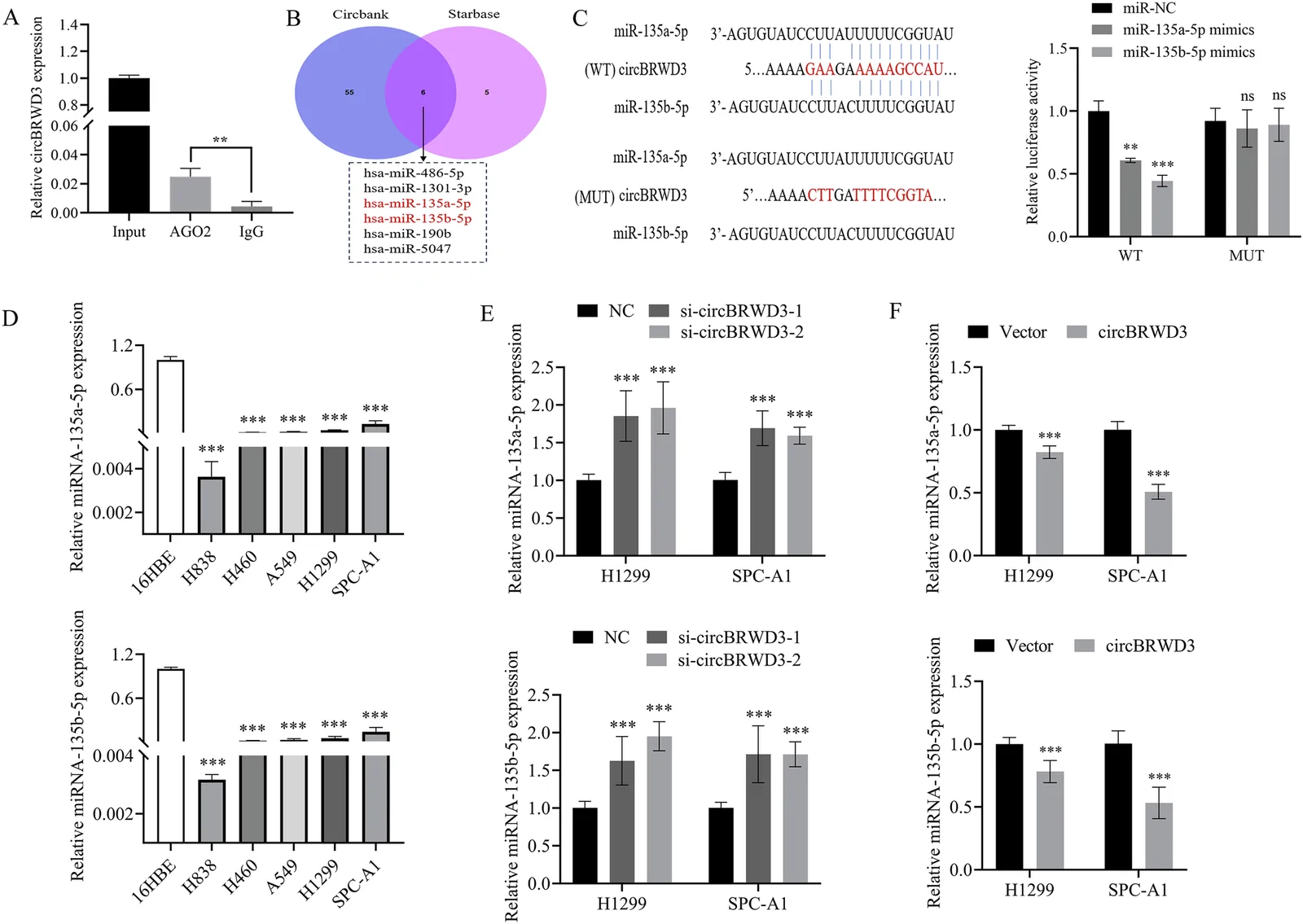
CircBRWD3 acted as a miRNA sponge for miR-135a-5p/miR-135b-5p. **A** RIP assay for the interaction of circBRWD3 with AGO2 protein. **B** The potential miRNAs were predicted by circBank and Starbase. **C** Luciferase reporter assay for luciferase activity of circBRWD3 in HEK293T cells co-transfected with miRNA mimics. **D** Relative expression of miR-135a-5p/miR-135b-5p in NSCLC cell lines. **E**, **F** qRT-PCR was applied to detect the expression level of miR-135a-5p/miR-135b-5p after circBRWD3 knockdown/overexpression in NSCLC cells.

Considering that circRNAs have been shown to regulate gene expression in cancer by acting as miRNA sponges, sequestering miRNAs to modulate the expression of their target genes [10, 22]. RNA FISH results showed that circBRWD3 was distributed in both the cytoplasm and nucleus of NSCLC cell lines (Fig. 1F), suggesting that circBRWD3 may be involved in the posttranscriptional regulation of circBRWD3.We then examined whether circBRWD3 could function as a miRNA sponge. RIP assay results showed that circBRWD3 was specifically enriched in beads containing AGO2 antibody compared with control IgG, suggesting that circBRWD3 could interact with AGO2 protein in NSCLC cells (Fig. 6A). We then predicted the potential circBRWD3-interacting miRNAs through bioinformatic databases (Circbank and Starbase). We identified six miRNAs (miR-486-5p, miR-1301-3p, miR-135a-5p, miR-135b-5p, miR-190b, miR-5047) that could potentially bind to circBRWD3 (Fig. 6B). The TCGA database revealed that the level of miR-135a-5p was lower in NSCLC tissues than in normal tissues. Then, we further identified a potential binding site in circBRWD3 for miR-135a-5p and miR-135b-5p. The results of dual-luciferase reporter assays showed that miR-135a-5p and miR-135b-5p mimics notably suppressed the luciferase activity of circBRWD3 wild-type reporter while not affected that of circBRWD3 mutant reporter (Fig. 6C). Additionally, qRT-PCR results also suggested that miR-135a-5p and miR-135b-5p were lower expression in NSCLC cells compared to 16HBE cells (Fig. 6D). Moreover, qRT-PCR analysis revealed that circBRWD3 knockdown led to an increase in the expression of miR-135a-5p and miR-135b-5p in NSCLC cells, whereas circBRWD3 overexpression resulted in a decrease in the expression of miR-135a-5p and miR-135b-5p (Fig. 6E, F). These findings suggested that circBRWD3 served as a sponge for miR-135a-5p and miR-135b-5p, leading to a decrease in its expression level.

### CircBRWD3 promoted the progression of NSCLC cells through sponging miR-135a-5p and miR-135b-5p

**Fig. 7:**
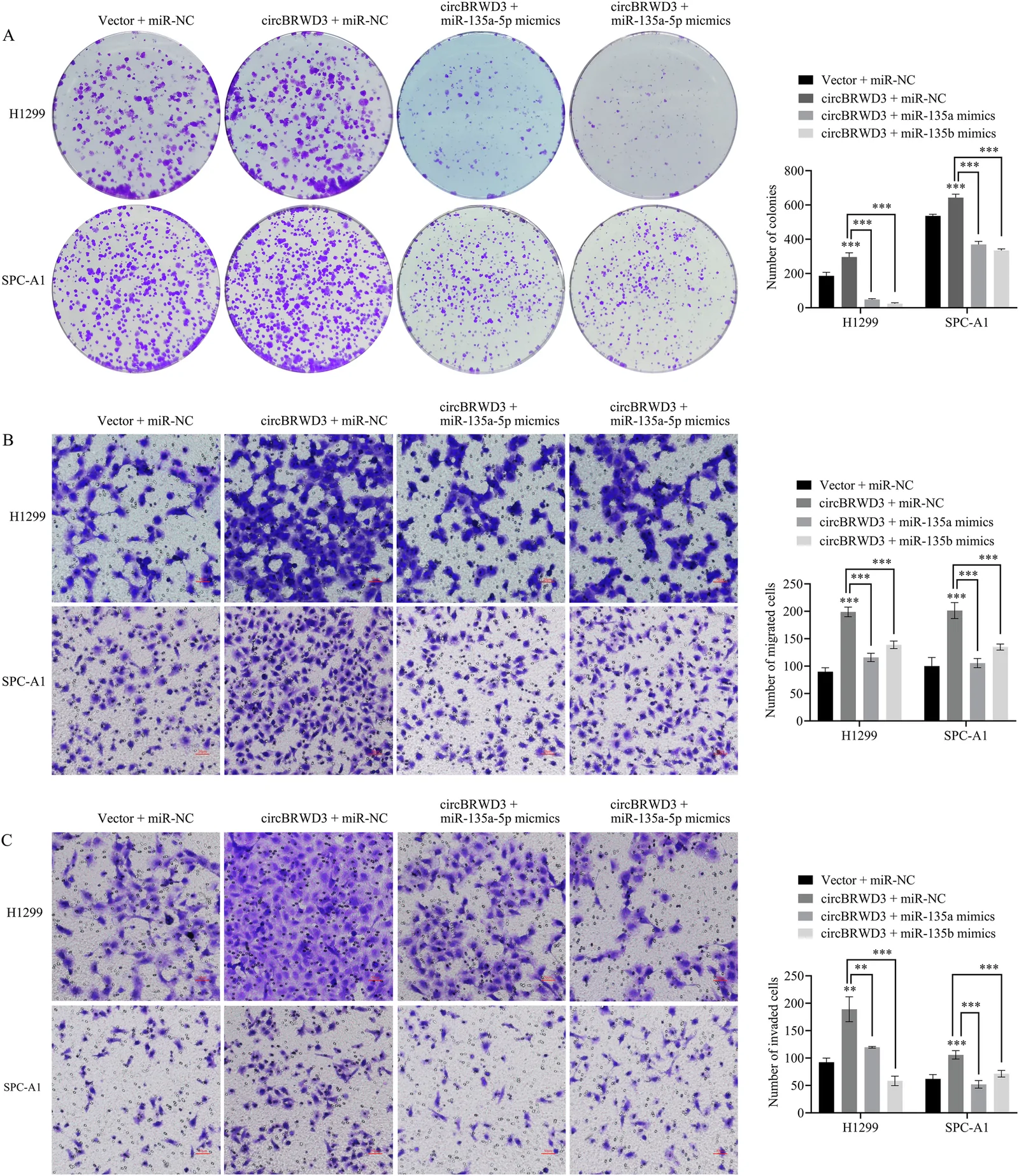
CircBRWD3 promoted the progression of NSCLC through sponging miR-135a-5p/miR-135b-5p. **A** The colony formation abilities of NSCLC cells after circBRWD3 overexpression or miR-135a-5p/miR-135b-5p mimics co-transfection. **B**, **C** The migration and invasion abilities of NSCLC cells.

We further determined the biological roles of miR-135a-5p and miR-135b-5p in NSCLC progression. To explore the importance of miR-135a-5p and miR-135b-5p in circBRWD3-regulated tumor promotion in NSCLC, miR-135a-5p and miR-135b-5p mimics were transfected into stable cells overexpressing circBRWD3. The further results showed that circBRWD3 overexpression promoted the proliferation, migration, and invasion of NSCLC cells, whereas co-transfection with miR-135a-5p and miR-135b-5p mimics attenuated these effects (Fig. 7A–C). These findings illustrated that circBRWD3 interacted with miR-135a-5p and miR-135b-5p to exert its oncogenic function in NSCLC.

### CircBRWD3-regulated miR-135a-5p/miR-135b-5p/SPTBN2 axis

**Fig. 8:**
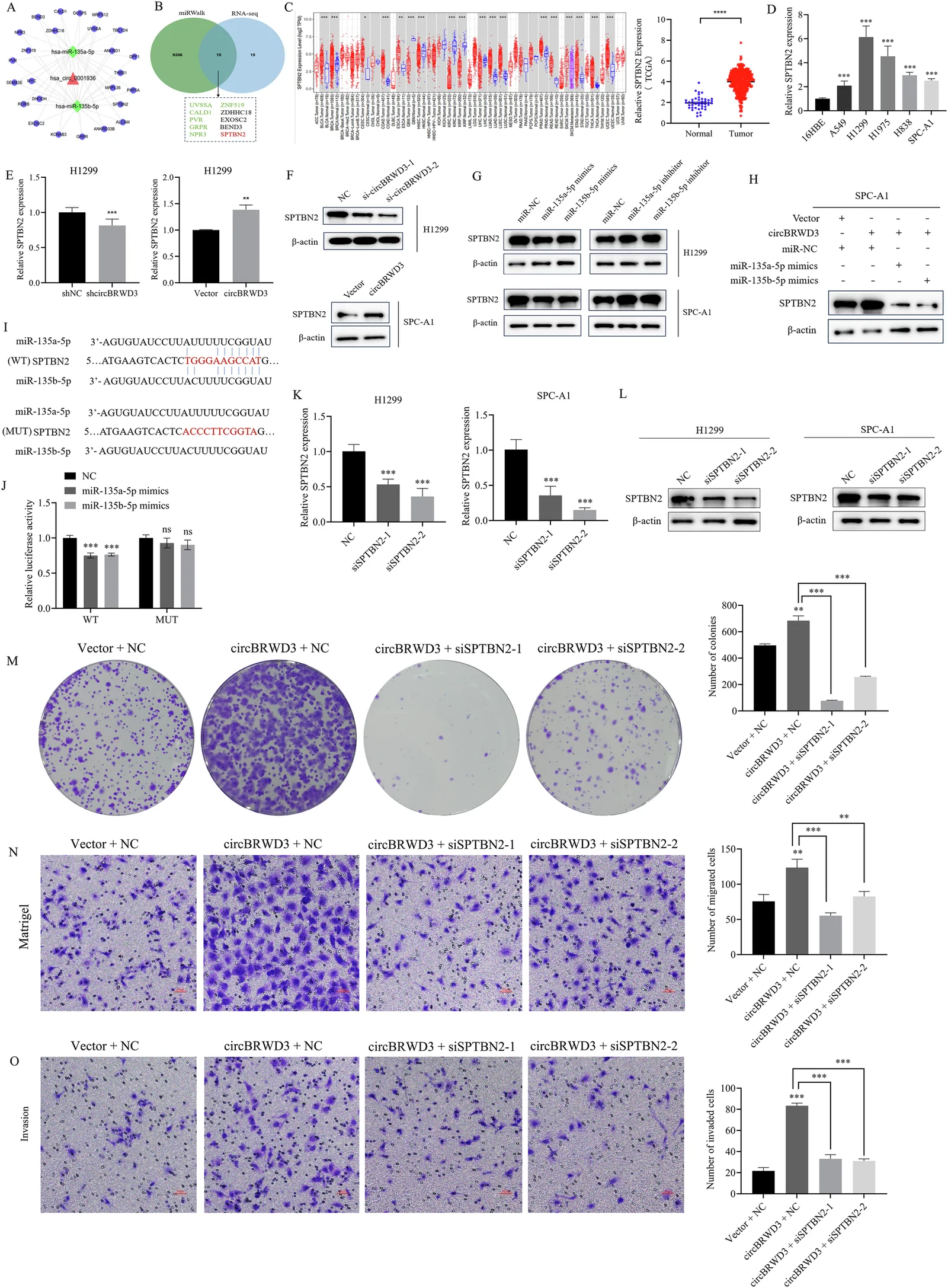
CircBRWD3-regulated miR-135a-5p/miR-135b-5p/SPTBN2 axis in NSCLC cells. **A** The potential target genes via bioinformatics analysis. **B** Identification of SPTBN2 as a candidate target of miR-135a-5p/miR-135b-5p by overlapping miRWalk predictions and RNA-seq data. **C** SPTBN2 expression of NSCLC in the TCGA database. **D** Relative expression of SPTBN2 in NSCLC cell lines. qRT-PCR (**E**) and western blot (**F**) were performed to detect the expression of SPTBN2 in NSCLC cells with circBRWD3 knockdown or overexpression. **G** A Western blot was performed to detect the expression of SPTBN2 in NSCLC cells transfected with miR-135a-5p/miR-135b-5p mimics or inhibitors. **H** The protein expression of SPTBN2 in circBRWD3 overexpression cells with co-transfection of miR-135a-5p or miR-135b-5p mimics was determined by western blot. **I**, **J** Relative luciferase activities of wild-type (WT) and mutated (MUT) SPTBN2 reporter plasmid in HEK293T cells co-transfected with miR-135a-5p or miR-135b-5p mimics. qRT-PCR (**K**) and western blot (**L**) analysis of SPTBN2 in NSCLC cells treated with two siRNAs against SPTBN2. Colony formation (**M**), transwell migration assay (**N**), and invasion assay (**O**) in NSCLC cells transfected with circBRWD3 overexpression and si-SPTBN2.

To identify potential target genes of miR-135a-5p and miR-135b-5p that may be regulated by circBRWD3, we analyzed the overlapped genes between miRWalk predictions and our RNA-seq data, selecting SPTBN2 as a downstream target for further investigation (Fig. 8A, B). SPTBN2 has been previously reported to function as an oncogene [23, 24]. Analysis of the TCGA database revealed that SPTBN2 mRNA level was significantly upregulated in LUAD tumor tissues compared to adjacent non-tumor tissues (Fig. 8C), which was further confirmed by qRT-PCR in NSCLC cell lines (Fig. 8D). Additionally, qRT-PCR results showed that the expression of the SPTBN2 gene decreased after circBRWD3 was knocked down, while the expression of the SPTBN2 gene increased after circBRWD3 was overexpressed (Fig. 8E). Meanwhile, western blot analysis also showed that a significant downregulation in SPTBN2 protein levels following transfection with si-circBRWD3, whares SPTBN2 protein level was obviously increased in circBRWD3-overexpression cells (Fig. 8F). The further results showed that SPTBN2 protein expression was significantly decreased following transfection with miR-135a-5p and miR-135b-5p mimics, compared to the respective controls. Whereas inhibition of miR-135a-5p and miR-135b-5p led to a marked upregulation of SPTBN2 protein levels in NSCLC cells (Fig. 8G). Interestingly, SPTBN2 protein levels were upregulated in the circBRWD3 overexpression cells but decreased in the miR-135a-5p or miR-135b-5p mimics group compared to those in the control group (Fig. 8H). Moreover, we verified the regulatory effect of miR-135a-5p and miR-135b-5p on SPTBN2 by using luciferase reporter assays. The results showed that miR-135a-5p and miR-135b-5p mimics significantly reduced luciferase activity when co-transfected with SPTBN2 wide-type (WT) 3′UTR, while this reduction was abrogated upon mutation of the predicted miR-135a-5p and miR-135b-5p binding sites (Fig. 8I, J). Thus, we explored the functional role of SPTBN2 in the circBRWD3/miR-135a-5p/miR-135b-5p axis in NSCLC progression. SPTBN2 was significantly downregulated in NSCLC cells transfected with si-SPTBN2 (Fig. 8K, L). SPTBN2 knockdown reversed the pro-tumorigenic effects of circBRWD3 overexpression, including enhanced NSCLC cell proliferation, migration and invasion (Fig. 8M–O). Taken together, these findings indicated that circBRWD3 acted as an oncogene in NSCLC progression via regulating the miR-135a-5p/miR-135b-5p/SPTBN2 axis.

## Discussion

NSCLC ranks among the most prevalent malignant tumors worldwide and is often associated with high rates of metastasis and recurrence. Although significant advances have been achieved in its diagnosis and treatment, particularly through surgery and chemotherapy, the prognosis for patients with advanced-stage NSCLC remains poor. Growing evidence suggests that circRNAs are differentially expressed in various pathological conditions, play vital regulatory roles in the initiation and progression of multiple cancers [25], including NSCLC [26], hepatocellular carcinoma [27], breast cancer [28], gastric cancer [29], and colorectal cancer [30]. CircRNAs can be utilized as promising biomarkers for cancer diagnosis and prognosis due to their high stability and specific loop structure. In this study, we focus on circBRWD3, which has previously not been reported in NSCLC, and was upregulated in tumor tissues and serum of NSCLC patients. We identified circBRWD3, a novel circRNA that was produced by back-splicing of the BRWD3 gene transcript, which was upregulated in tumor tissues and its high expression was associated with tumor size in NSCLC patients. We next explored the molecular roles and regulatory pathways through which circBRWD3 drives NSCLC progression. During our study in preparation, another study by Meng et al. reported that circBRWD3 expression was dramatically upregulated in breast cancer tissues, a feature that was positively correlated with the poor prognosis of patients with breast cancer. They confirmed that circBRWD3 facilitates breast cancer tumorigenesis by regulating the circBRWD3/miR-142-3p_miR-142-5p/RAC1/PAK1 axis [31]. Our findings are consistent with this study, indicating that circBRWD3 upregulation may be linked to the progression of different cancers.

Previous studies have identified various circRNAs that are dysregulated in NSCLC. For instance, circPOLA2 is highly expressed in lung cancer tissues and predicts poor prognosis by promoting cancer cell stemness and progression through the miR-326/GNB1 axis [10]. CircHERC1 was significantly upregulated in lung cancer tissues and cells, and facilitates the invasion and metastasis of NSCLC cells by regulating the miR-142-3p/HMGB1 axis and activating the MAPK/ERK and NF-κB pathways [9]. CircSORBS1 is significantly downregulated in lung cancer tissues, blood, and cell lines, and its low expression is associated with poor prognosis. Functionally, circSORBS1 suppresses lung cancer progression by sponging miR-6779-5p and stabilizing RUFY3 mRNA, thereby activating the YWHAE/BAD/BCL2 apoptotic signaling pathway [32]. Circ_0078767 was downregulated in NSCLC tissues, and its upregulation inhibited NSCLC cell proliferation, cell cycle progression and EMT while inducing cell apoptosis [33]. In this study, we revealed the biological roles of circBRWD3 in NSCLC both in vitro and in vivo. Mechanistically, circBRWD3 acted as a ceRNA to regulate the miR‑135a‑5p/miR‑135b‑5p/SPTBN2 axis in NSCLC cells. Our findings indicated that circBRWD3 may function as an oncogene circRNA to promote NSCLC progression, which may enhance our understanding of the roles of circBRWD3 in NSCLC pathogenesis and highlight its potential as a biomarker and therapeutic target. In the last 10 years, the study of circular RNAs has matured into a recognized discipline, clarifying how these distinct transcripts influence tumor biology [25]. CircRNAs have been shown to exert biological functions by acting as microRNA sponges, RBP sponges, regulators of gene splicing and transcription, and protein/peptide translators [34]. Studies have found that circPPAP2B in clear cell renal cell carcinoma (ccRCC) tissues, particularly in metastatic ccRCC tissues, and found it to be associated with poor prognosis. circPPAP2B interacts with HNRNPC in a m6A-dependent manner to facilitate HNRNPC nuclear translocation and functions as a miRNA sponge to directly bind to miR-182-5p and increase CYP1B1 expression in ccRCC to promote proliferation and metastasis [18]. Gastric-cancer-cell-derived exosomal circATP8A1 drives M2 macrophage polarization and tumor progression via the circATP8A1/miR-1-3p/STAT6 axis, positioning circATP8A1 as a prognostic biomarker and therapeutic target in gastric cancer [29]. circPOLR2A modulated the UBE3C-mediated ubiquitination and degradation of the PEBP1 protein, and further activated the ERK pathway during clear cell renal cell carcinoma (cRCC) progression and metastasis [35]. A recent study revealed that circRANBP17 interacts with the RBP UPF1 to form a complex that accelerates the degradation of SIRT6 mRNA, thereby perturbing lipid metabolism and promoting lung adenocarcinoma progression [36]. Studies have found that circPRKCA engages in interaction with Y-box binding protein 1 (YBX1) within the cytoplasmic milieu, consequently preventing the ubiquitination-mediated degradation of YBX1 to enhance the capabilities of migration, invasion, and angiogenesis in esophageal squamous cell carcinoma (ESCC) [37]. CircCAPRIN1 interacted with STAT2 to promote CRC tumor progression and lipid synthesis by enhancing the expression of ACC1 [16]. CircNFATC3 bound to IGF2BP3 in the cytoplasm, which enhanced the stability of IGF2BP3 by preventing ubiquitin E3 ligase TRIM25-mediated ubiquitination, thereby enhancing the regulatory axis of IGF2BP3-CCND1 and promoting CCND1 mRNA stability [38]. In colorectal cancer, reduced circLPAR1 expression predicts poor prognosis, tumor-suppressive exosomal circLPAR1 is taken up by CRC cells, and it suppresses tumor growth, likely because exosomal circLPAR1 directly binds with eIF3h, specifically suppressing the METTL3-eIF3h interaction, decreasing the translation of oncogene BRD4 [39]. Notably, RBPs such as IGF2BP3 can also be targeted by small molecules to modulate RNA stability; baicalin has been shown to disrupt the m⁶A-IGF2BP3-LDHA axis, leading to reduced LDHA mRNA stability and inhibition of tumor progression.

An increasing number of studies have shown that circRNAs contain miRNA response elements and function as miRNA sponges, a mechanism extensively investigated in cancer [25, 40]. For example, CircRNA_104348 might function as a competing endogenous RNA to promote hepatocellular carcinoma progression by targeting miR-187-3p/RTKN2 axis and activating Wnt/β-catenin pathway [41]. CircBCBM1 acted as an endogenous miR-125a sponge to upregulate the expression of BRD4 (bromodomain containing 4) and MMP9 (matrix metallopeptidase 9) through Sonic hedgehog (SHH) signaling pathway to promote breast cancer growth and metastasis [42]. CircRNA_0008315 interacted with miR-3666 to upregulate CPEB4 to modulate the stem cell properties of GC, exacerbated GC progression and cisplatin resistance [43]. The study demonstrated that circPPAP2B functions as a miRNA sponge to directly bind to miR-182-5p and increase CYP1B1 expression in clear cell renal cell carcinoma [18]. Circ_0001666 functions as an oncogenic circRNA in pancreatic cancer by sponging miR-1251, thereby upregulating its downstream target SOX4 and promoting EMT and cell invasion [44]. The latest research has found that circSLC39A10 has been reported to act as a molecular sponge for miR-936 in prostate cancer, releasing its target PROX1 to activate the Wnt/β-catenin signaling pathway and promote tumor proliferation and metastasis [45]. The circRNA-miRNA-mRNA regulatory axis also plays an important role in NSCLC. For instance, circZNF707 can upregulate PFKM by acting as a sponge for miR-668-3p, thus contributing to enhance proliferation, migration, invasion, and glycolysis of NSCLC cells [46]. circNOX4 interacted with miR-329-5p to upregulate the expression of FAP to promote NSCLC progression [26]. The subcellular localization of the circBRWD3 is mainly located in the cytoplasm, and potentially functions as a miRNA sponge. To test this hypothesis, luciferase activity assays have shown that circBRWD3 negatively regulates miR-135a-5p/miR-135b-5p. MiR-135a-5p was diminished in gastric cancer specimens and cells [47]. The expression level of miR-135a-5p was significantly reduced in CRC tissues as compared to that in normal tissues. The overexpression of miR-135a-5p using miR-135a-5p mimics significantly inhibited the proliferation, migration, invasion and promoted apoptosis of colorectal cancer cells [48]. MiR-135a-5p expression was significantly decreased in lung cancer, lung SCC, and lung adenocarcinoma tissues compared to their respective adjacent normal tissues, and inhibited proliferation, migration, and invasion of NSCLC cells as well as enhanced apoptosis by the STAT6 signaling pathway [49]. In gallbladder cancer, miR-135a-5p affected GBC cell proliferation and invasion by targeting ANGPT2 [50]. Our findings revealed that miR-135a-5p/miR-135b-5p was downregulated, and its expression level is negatively correlated with circBRWD3 in NSCLC cells. The dual-luciferase reporter gene assay showed that circBRWD3 contained binding sites for miR-135a-5p/miR-135b-5p. The results indicated that miR-135a-5p/miR-135b-5p might also have an anti-tumor role by inhibiting the proliferation, migration, invasion and EMT of NSCLC cells. We demonstrated that circBRWD3 overexpression promoted NSCLC cell proliferation, migration, and invasion and that miR-135-5p co-transfection partially rescued the suppressive effect of circBRWD3 on NSCLC progression, indicating that circBRWD3 may serve as a ceRNA of miR-135a-5p in NSCLC.

Studies have indicated that circular RNAs can sponge microRNAs, thereby modulating the expression of their downstream target mRNAs by targeting the 3′-untranslated regions, and thus promoting tumorigenesis [51, 52]. CircCHSY1 acts as a competitive endogenous RNA that sequesters miR-24-3p, de-repressing the 3′-UTR of HO-1 mRNA. This microRNA sponging elevates HO-1 protein, thereby exerting protective effects that counteract tumorigenic stress responses [53]. CircRNF13 binds the 3′-UTR of SUMO2 mRNA to stabilize its transcript, elevating SUMO2 protein that SUMOylates and ubiquitinates GLUT1 for degradation, thereby suppressing glycolysis and halting nasopharyngeal carcinogenesis cells’ proliferation and metastasis [54]. Thus, the circRNF13–SUMO2–GLUT1 axis constitutes a critical metabolic brake in nasopharyngeal carcinogenesis. CircBCAR3 promoted the proliferation, migration and invasion of esophageal cancer cells by binding with miR-27a-3p to upregulate TNPO1 expression [55]. In this study, we have identified that circBRWD3 interacts with miR-135a-5p/miR-135b-5p to upregulate SPTBN2 to promote cell growth and migration in NSCLC. SPTBN2 was identified as a tumor-promoting gene in many cancers, and its upregulation was positively associated with poor prognosis [56, 57]. It was proposed to promote tumor cell proliferation, the cell cycle, migration, colony formation, and invasion and suppress cell apoptosis [58]. SPTBN2 was highly expressed in colorectal cancer tissues and cells. In ovarian cancer, SPTBN2 regulates endometroid cell proliferation, invasion and migration via ITGB4-mediated focal adhesion and extracellular matrix receptor signaling pathway [23]. Studies found that inhibiting SPTBN2 increased the sensitivity of NSCLC cells to cisplatin through ferroptosis induction. SPTBN2 suppressed ferroptosis in NSCLC cells by facilitating SLC7A11 membrane trafficking and localization [24]. In this study, our results showed that real-time PCR and western blot assays showed that the inhibition of miR-135a-5p and miR-135b-5p remarkably enhanced the levels of SPTBN2 in H1299 and SPC-A1 cells. Luciferase reporter assay showed that miR-135a-5p/miR-135b-5p could directly bind to the 3′UTR of SPTBN2 and negatively regulate SPTBN2 transcription. Moreover, the proliferation and metastatic roles of circBRWD3 overexpression on NSCLC cells can be weakened by co-transfection with SPTBN2 siRNA. Consistent with these studies, these findings suggested that circBRWD3 promoted the proliferation, migration and invasion in NSCLC via regulating the miR-135a-5p/miR-135b-5p/SPTBN2 axis.

This study has certain constraints. Although the expression of circBRWD3 was found to be high in NSCLC, the expression of circBRWD3 has not been thoroughly investigated in other tumors. In addition, circBRWD3 may exert its oncogenic functions through multiple regulatory mechanisms in NSCLC. In the present study, we verified that circBRWD3 promotes NSCLC progression not only by interacting with TRIM25 protein and enhancing its stability to mediate the ubiquitin-dependent degradation of p53, but also by acting as a ceRNA to regulate the miR-135a-5p/miR-135b-5p/SPTBN2 axis. Nevertheless, the comprehensive regulatory network of circBRWD3 in NSCLC remains to be further elucidated, particularly regarding the downstream biological consequences of the circBRWD3/SPTBN2 axis in broader tumor biology. Given that SPTBN2 is known to suppress ferroptosis in NSCLC cells by facilitating SLC7A11 membrane trafficking and localization [24], it is plausible that circBRWD3 may further impinge on metabolic homeostasis within the tumor microenvironment. This is particularly relevant, as a recent study highlighted that targeting the m⁶A-IGF2BP3-LDHA axis to reduce lactate accumulation potently reverses immune escape by enhancing CD8⁺ T-cell infiltration and inhibiting PD-L1 in oral squamous cell carcinoma [59]. Thus, future studies are needed to determine whether circBRWD3 exerts similar effects on the metabolic-immune axis in NSCLC, which would significantly expand our understanding of its oncogenic functions. Moreover, whether NSCLC patients with high circBRWD3 expression could benefit from conventional chemotherapy or targeted therapies warrants further investigation.

## Conclusion

**Fig. 9:**
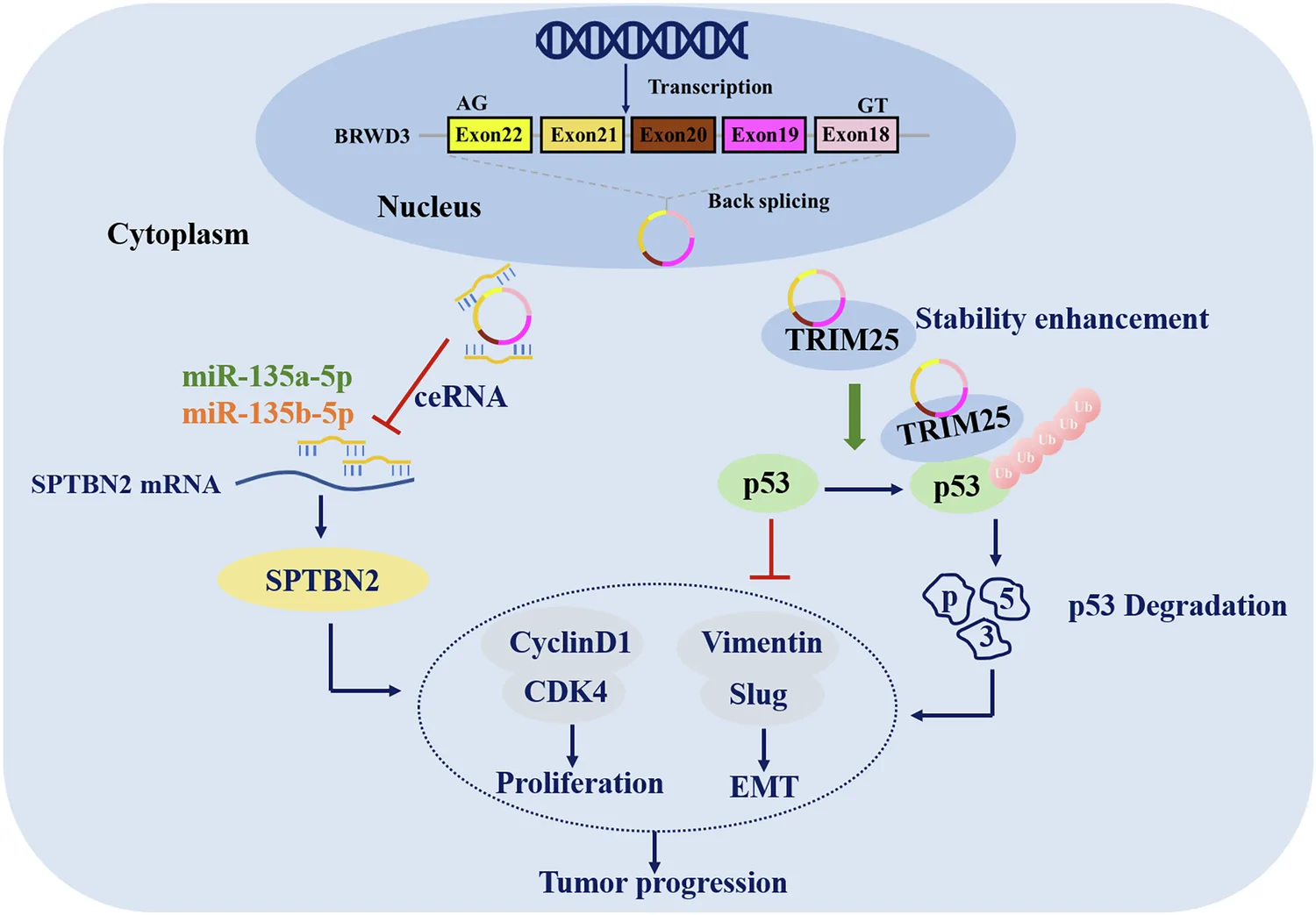
The schedule diagram of the circBRWD3 function.

In summary, our study revealed that circBRWD3 is upregulated in NSCLC progression and elucidated its mechanism of action (Fig. 9). We have found that circBRWD3 was upregulated in NSCLC tissues and serum. CircBRWD3 has the potential to influence cell growth and metastasis. CircBRWD3 enhanced the stability of TRIM25 to mediate ubiquitination and proteosomal degradation of p53 protein. In addition, circBRWD3 functioned as a sponge of miR-135a-5p/miR-135b-5p, and in turn promoted the expression of SPTBN2. Therefore, these findings revealed that circBRWD3 held promise as a biomarker for the diagnosis and treatment of NSCLC.

## Supplementary information

Original Images for Western blot

## Data Availability

All data and materials in this paper are available upon request.
