# Supplementary material for: CircBRWD3 promotes non-small cell lung cancer progression through enhancing the stability of TRIM25 to increase p53 ubiquitin degradation and upregulating SPTBN2
Source: Cell Death Dis. 2026 Jul 16;17(1):878. doi: 10.1038/s41419-026-09103-3 (PMC13655122; doi:10.1038/s41419-026-09103-3)

Original Images for Figure 4B

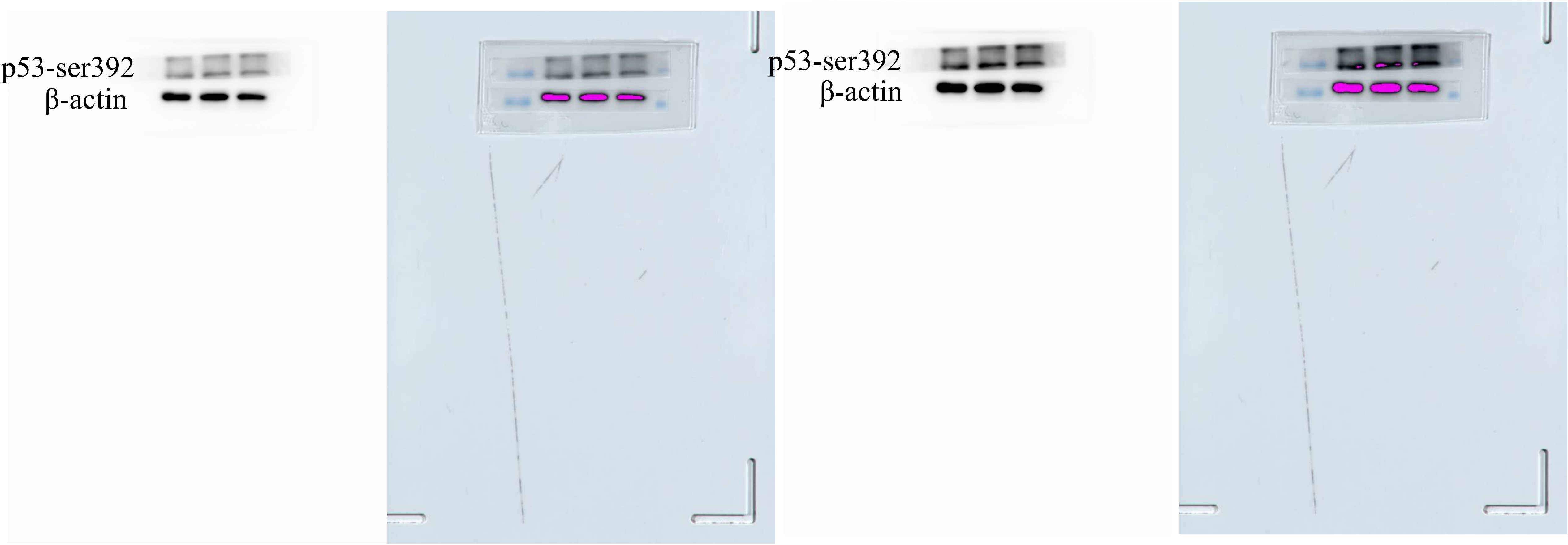

Original Images for Figure 4B

p53-ser15
β-actin

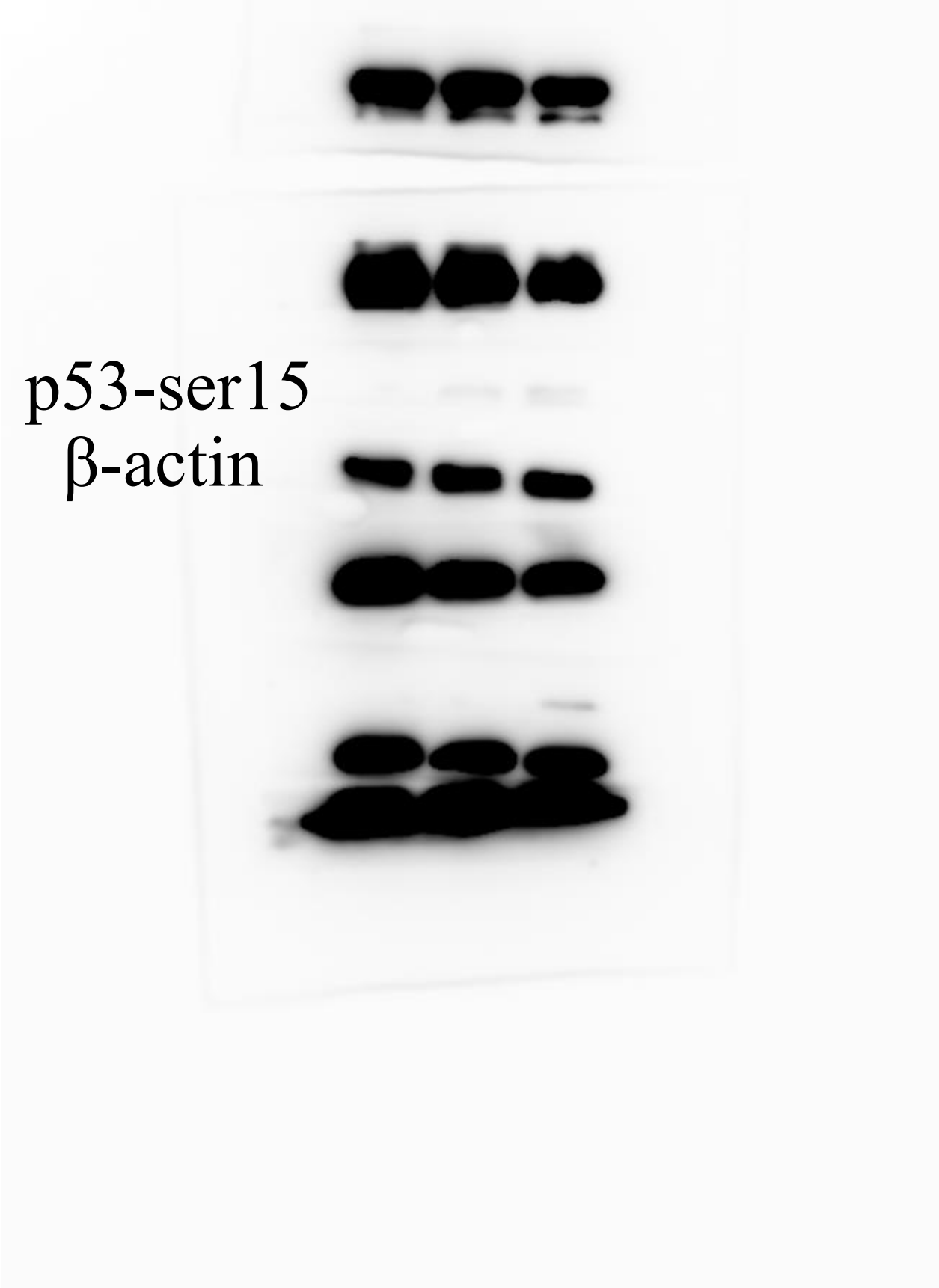

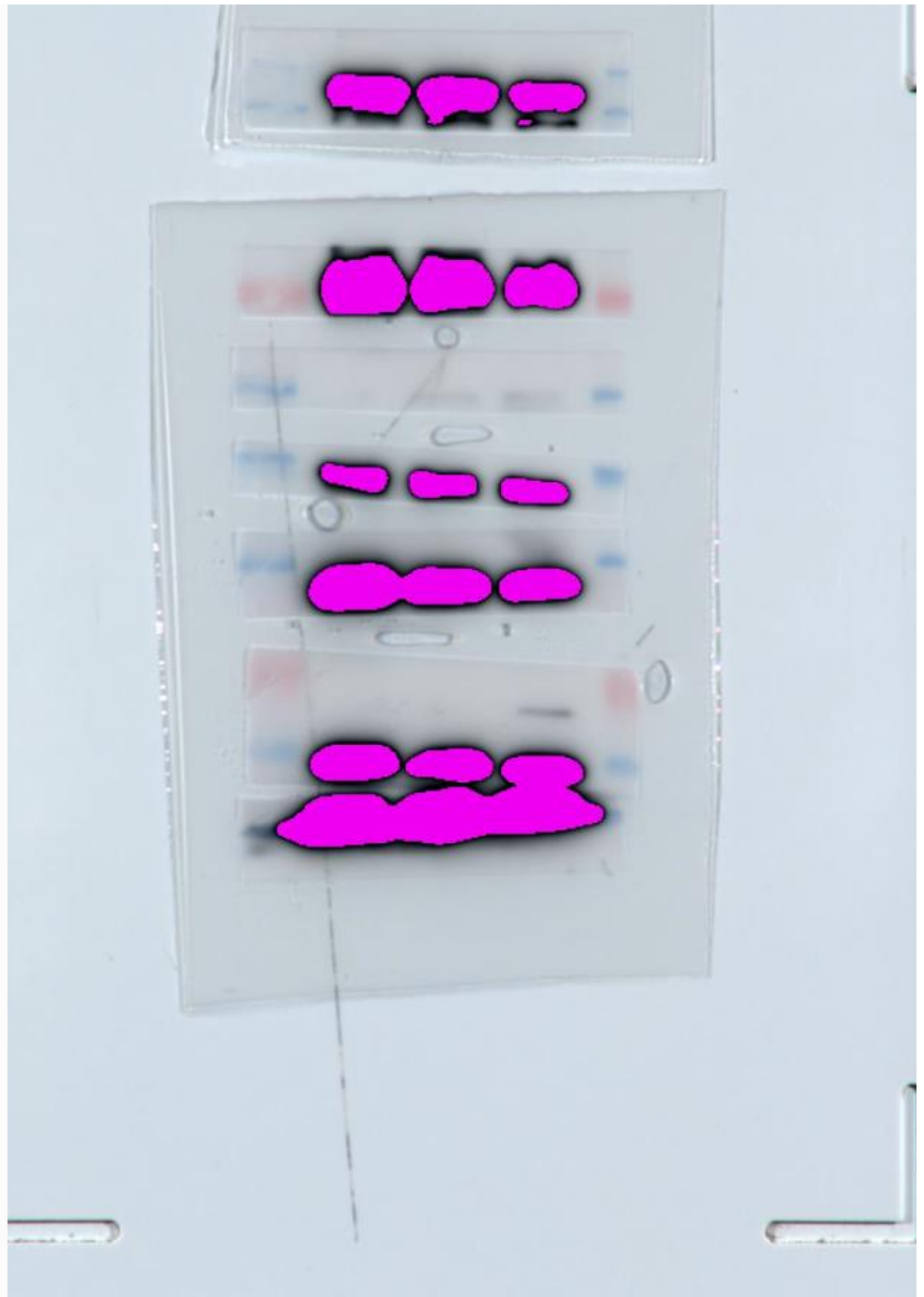

p53-ser15
β-actin

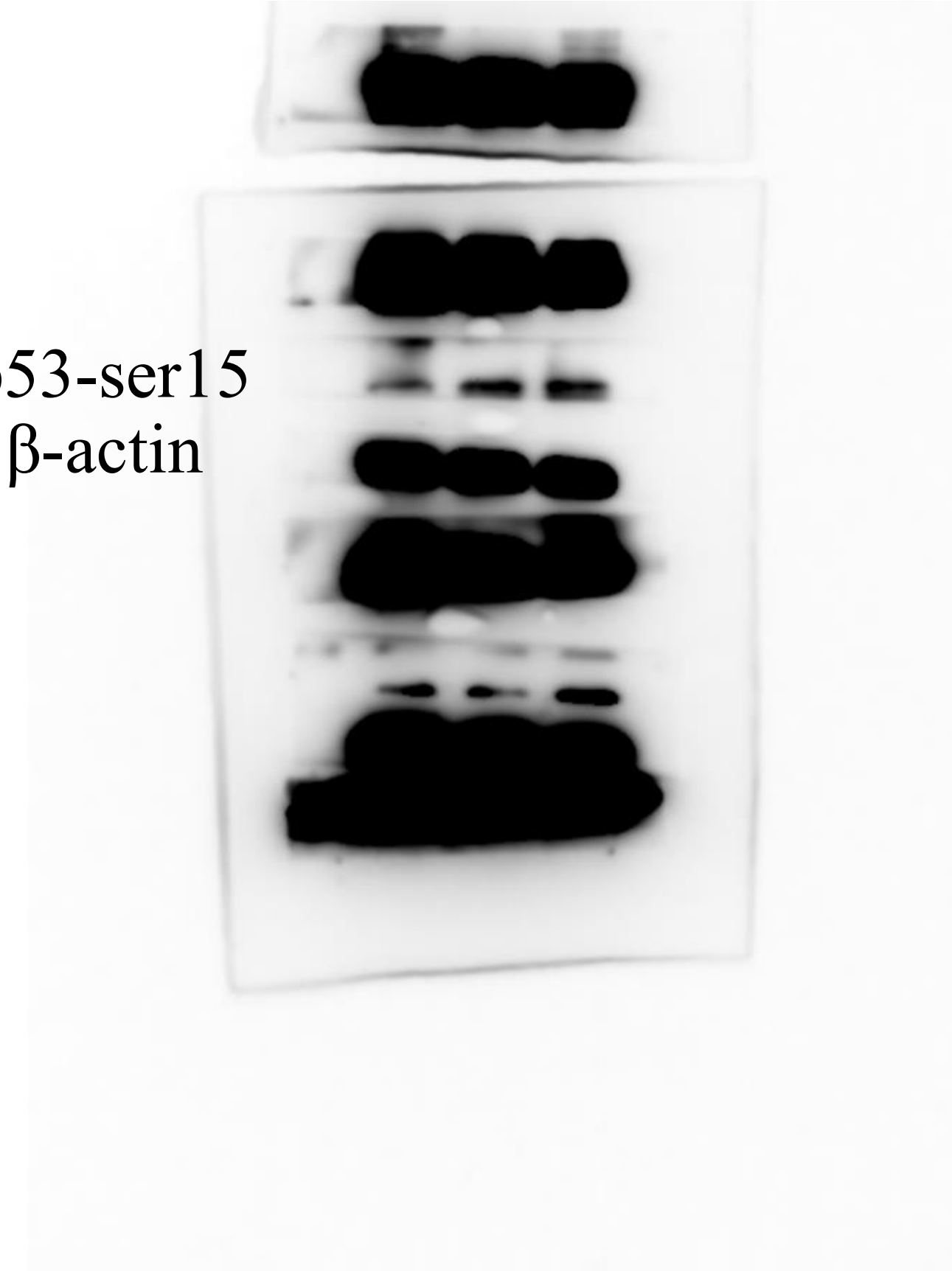

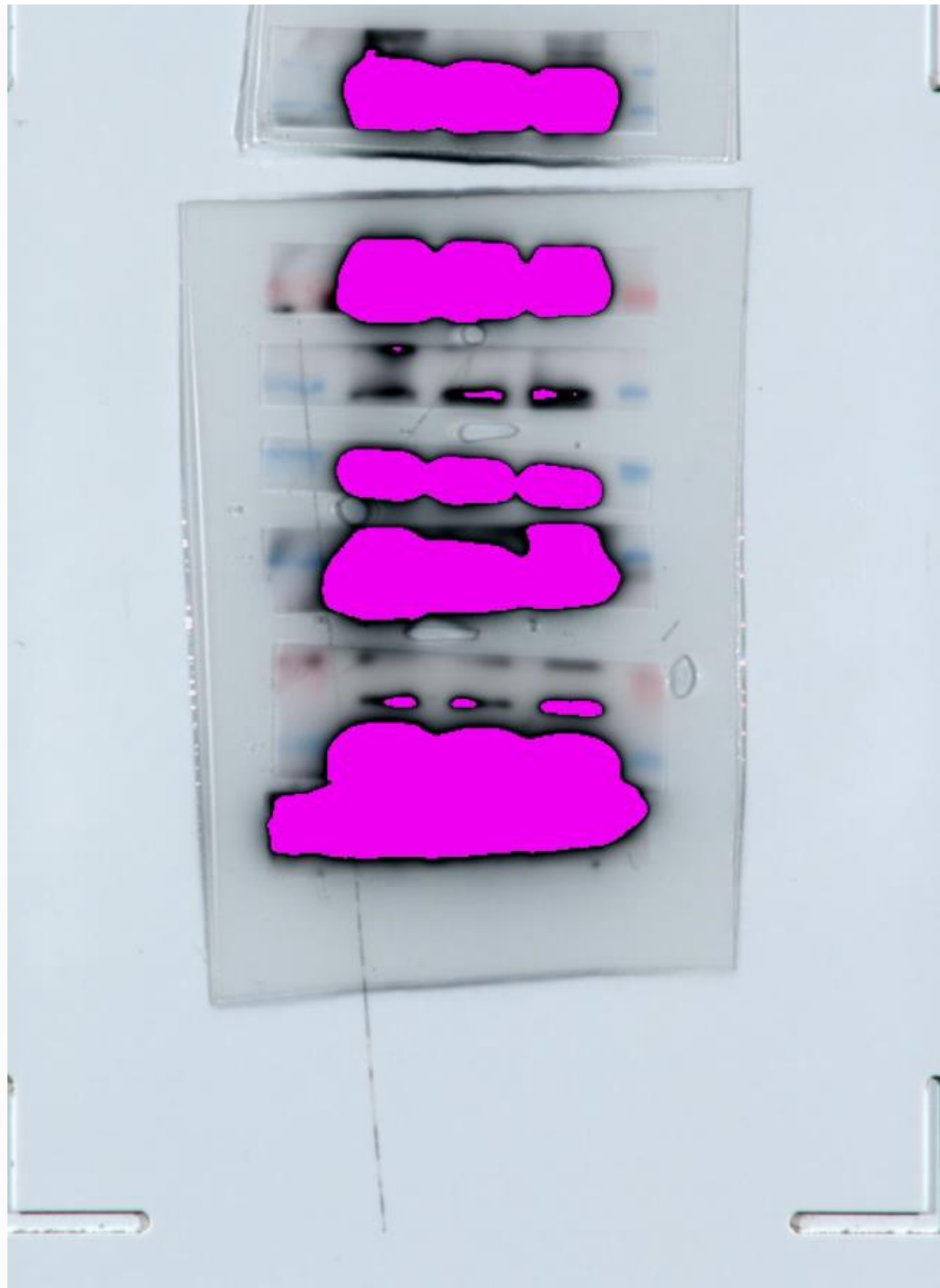

Original Images for Figure 4B

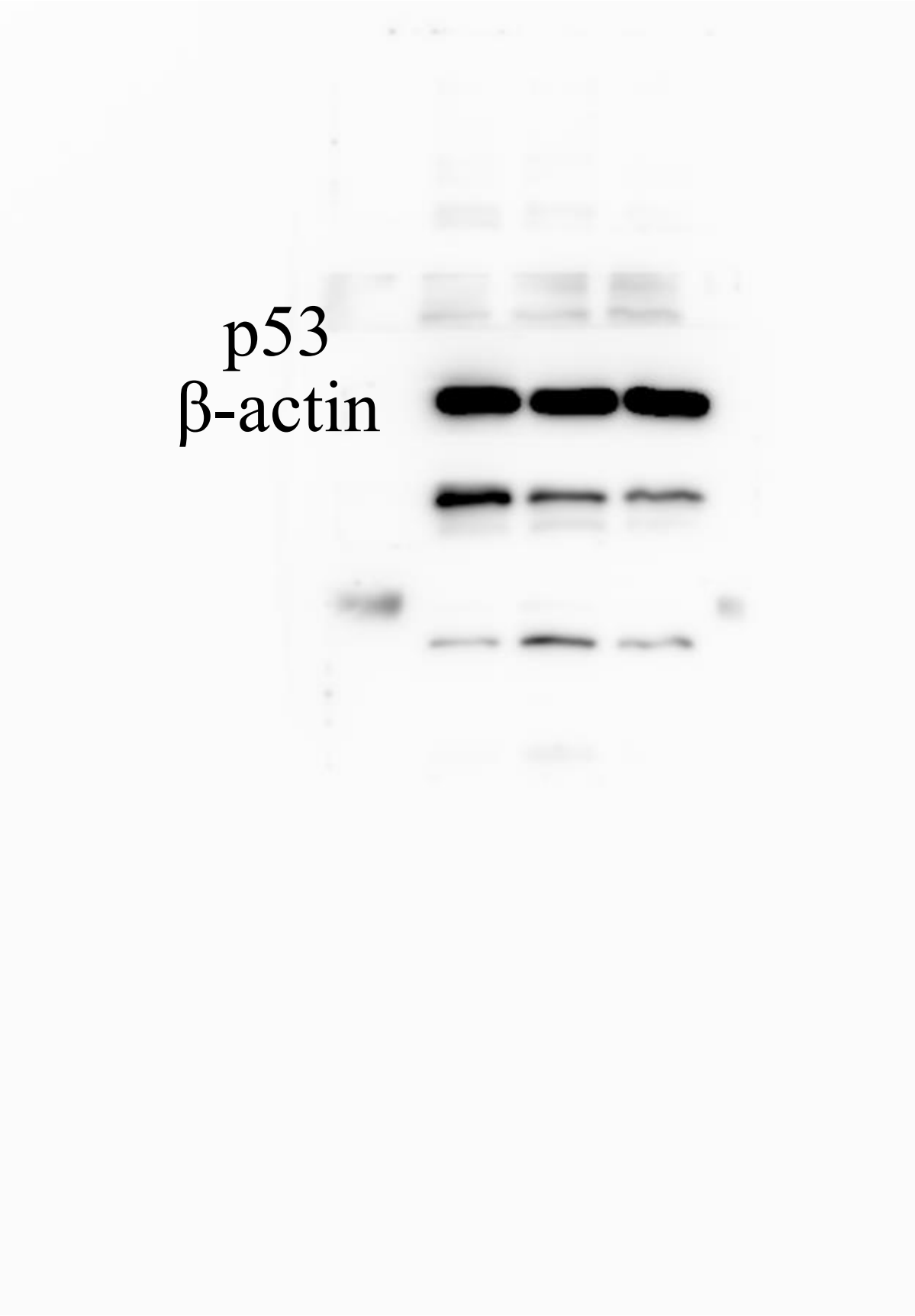

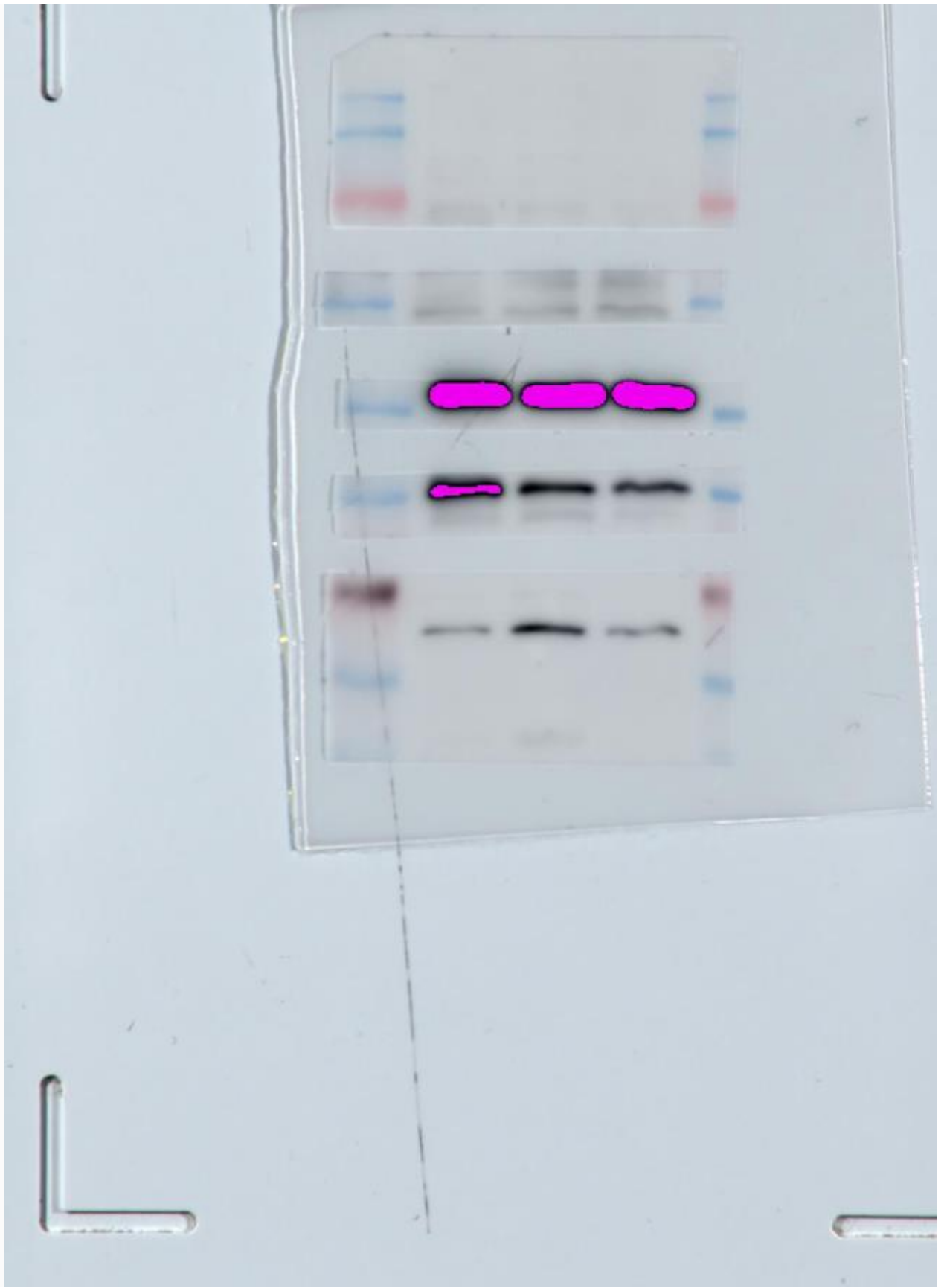

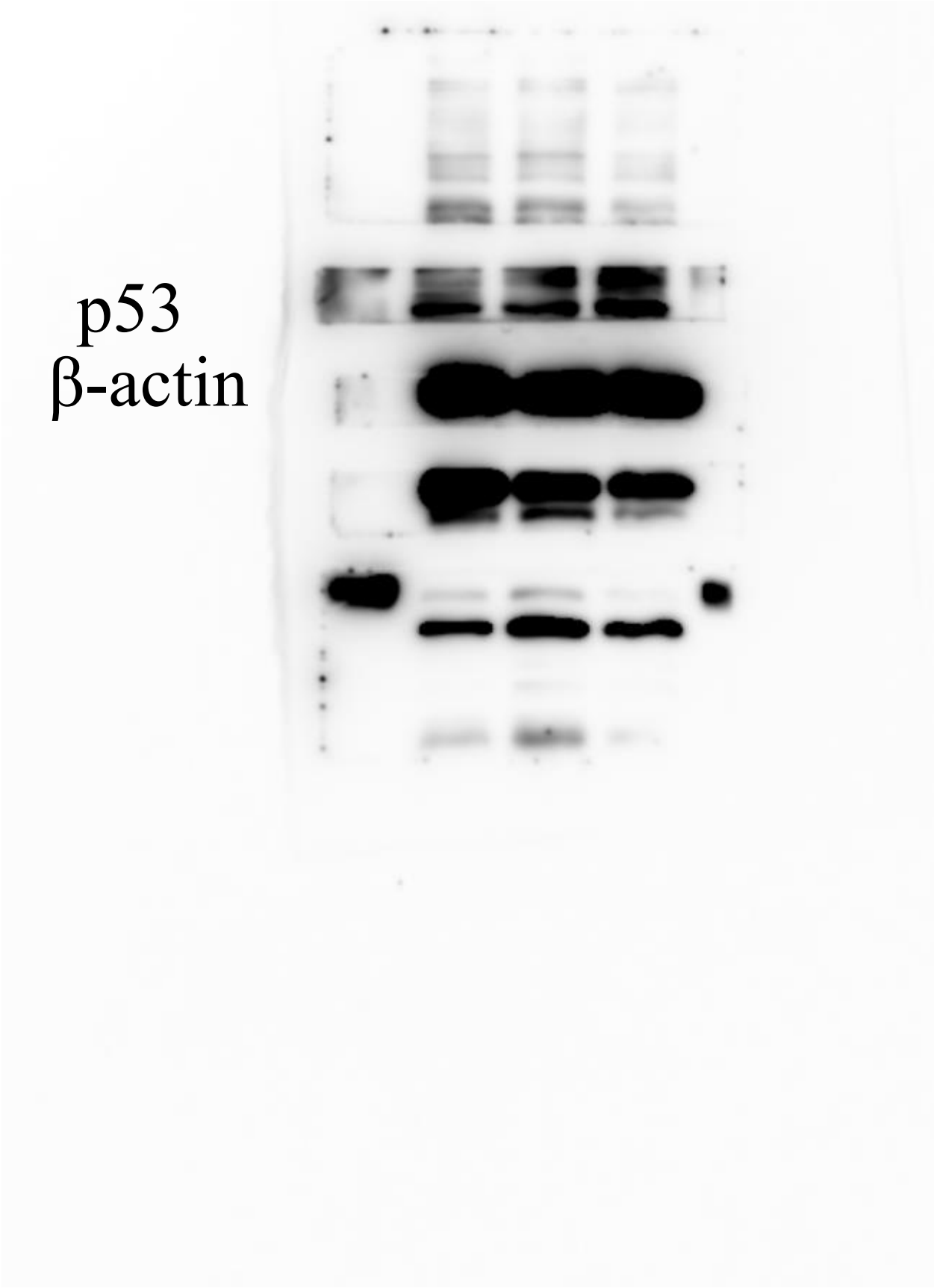

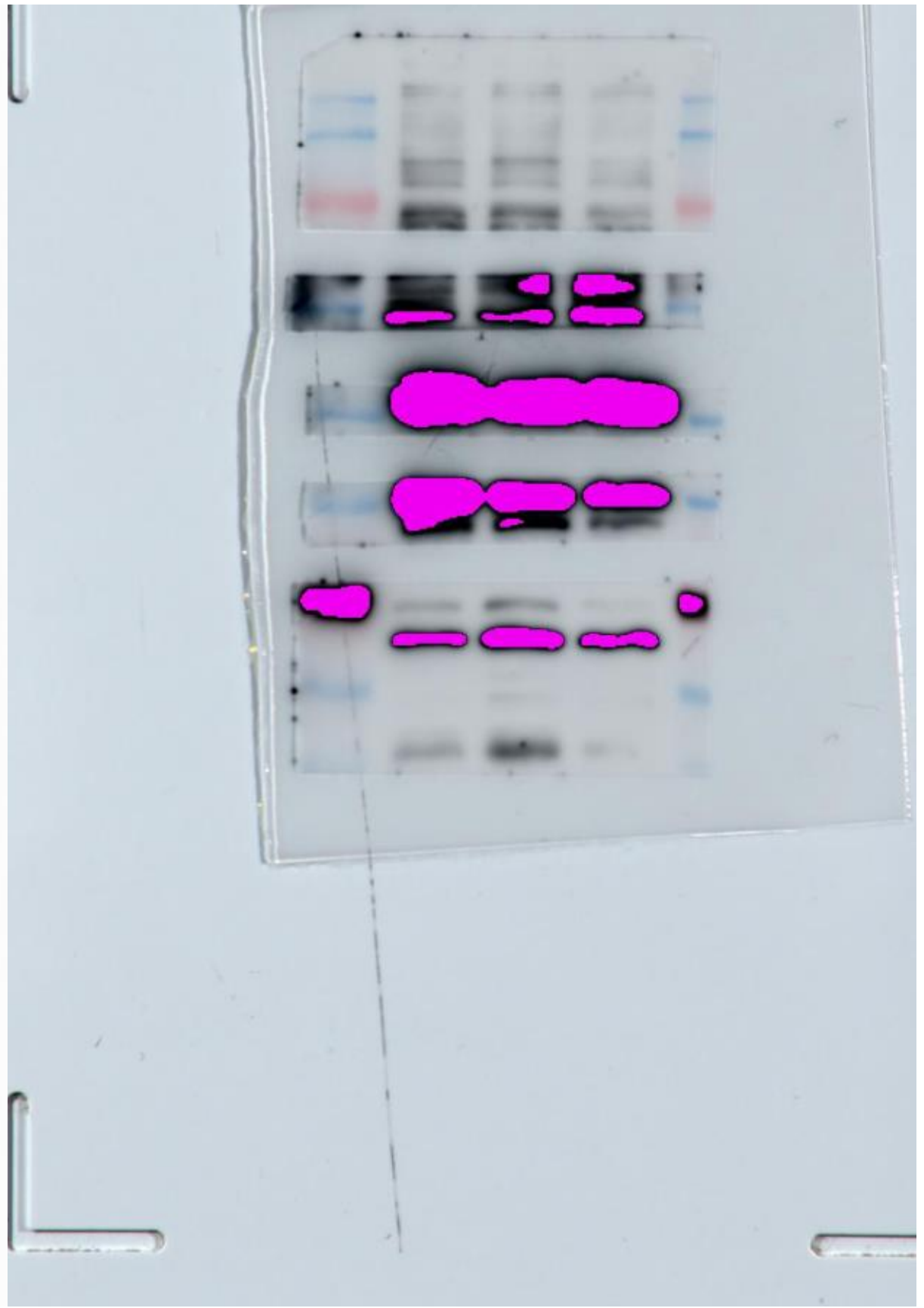

Original Images for Figure 4C

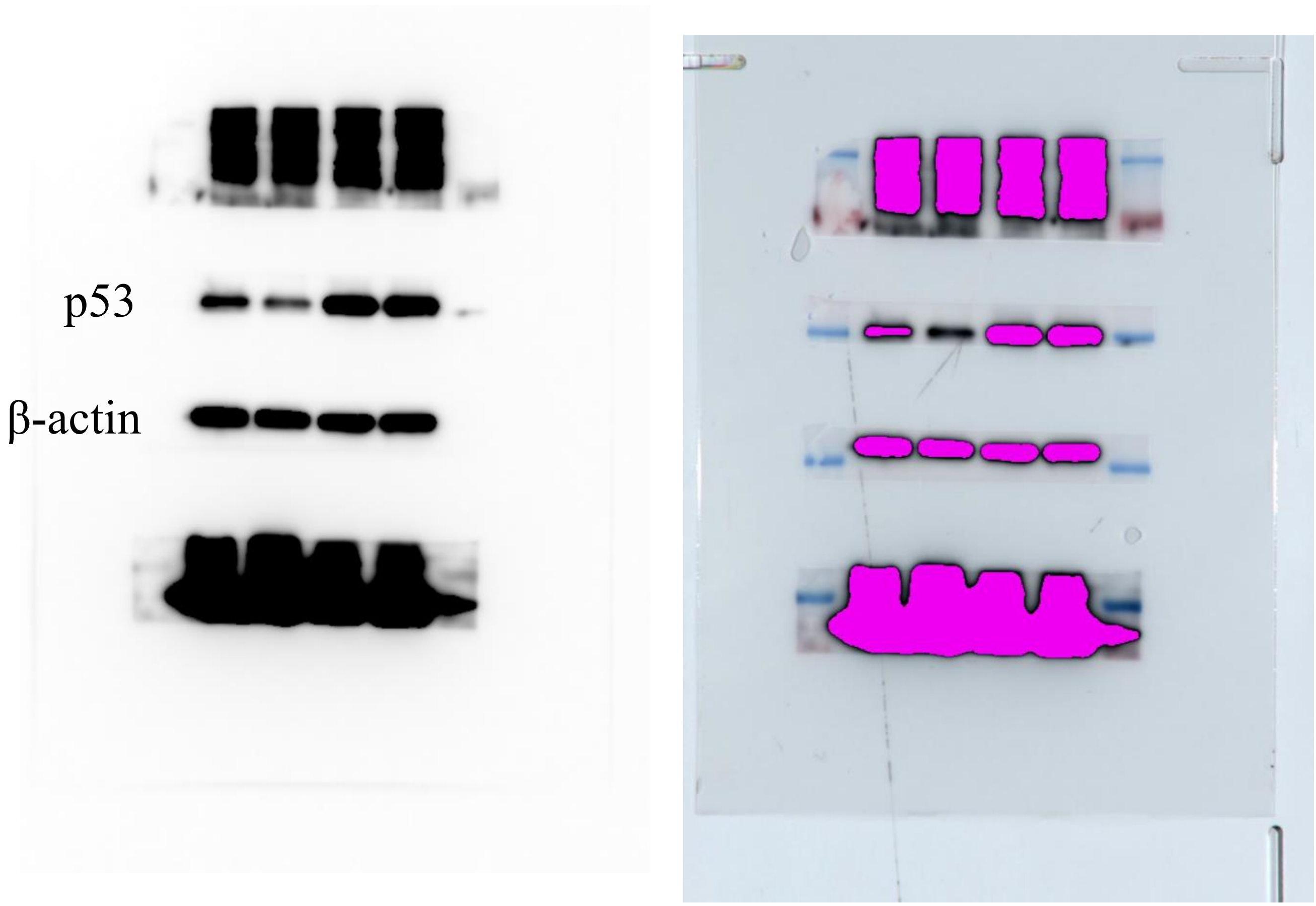

Original Images for Figure 4D

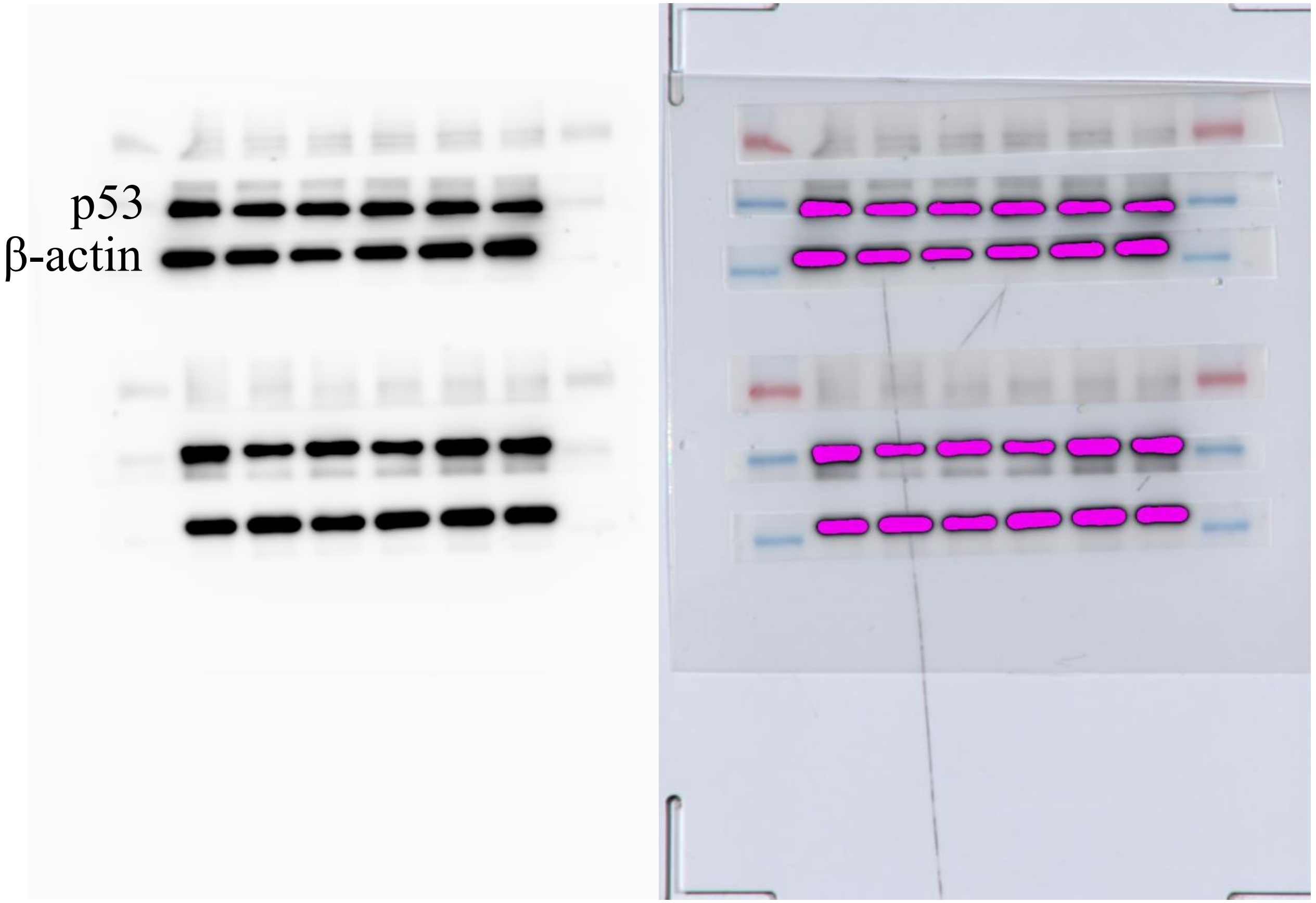

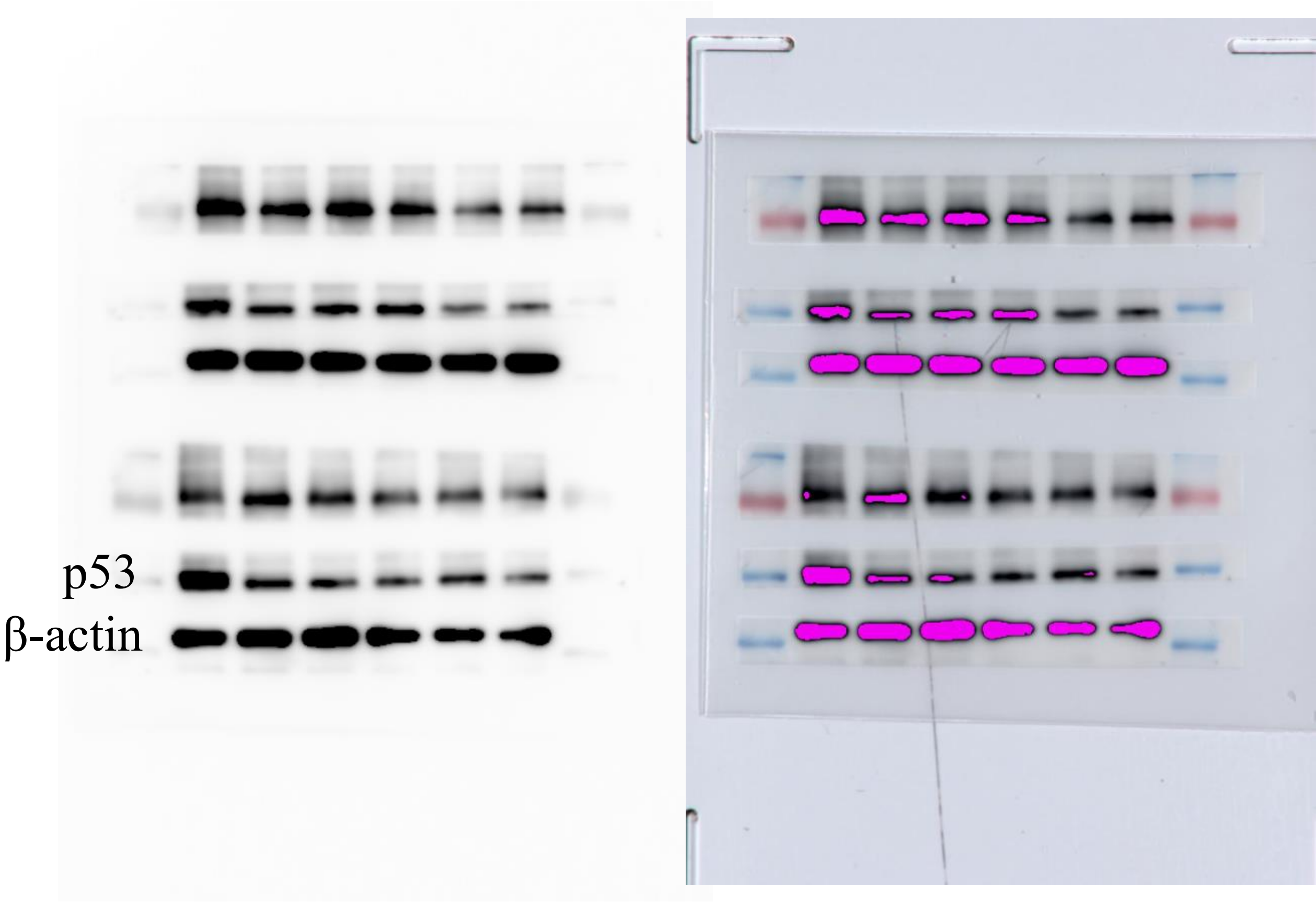

Original Images for Figure 4E

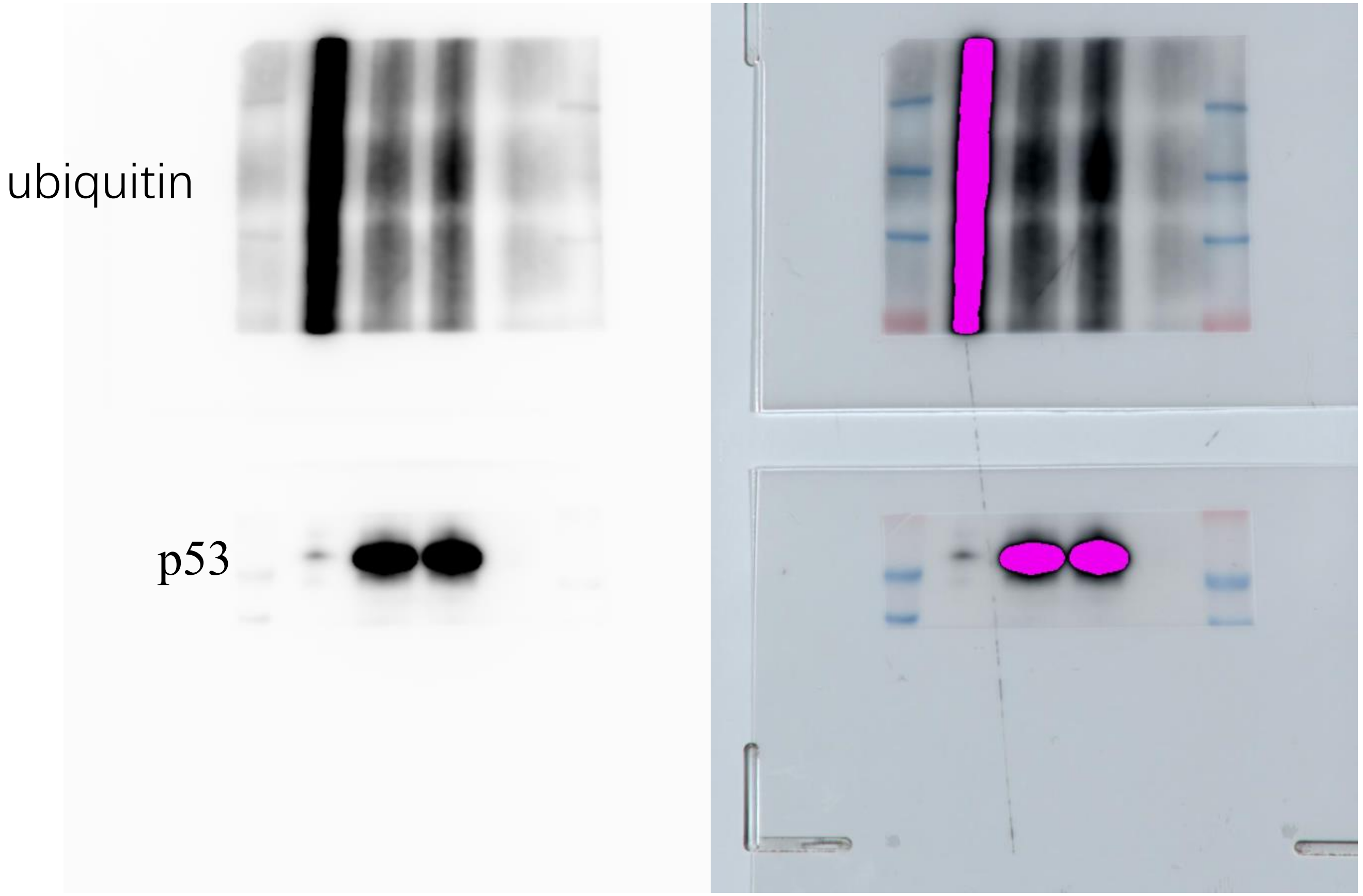

Original Images for Figure 4G

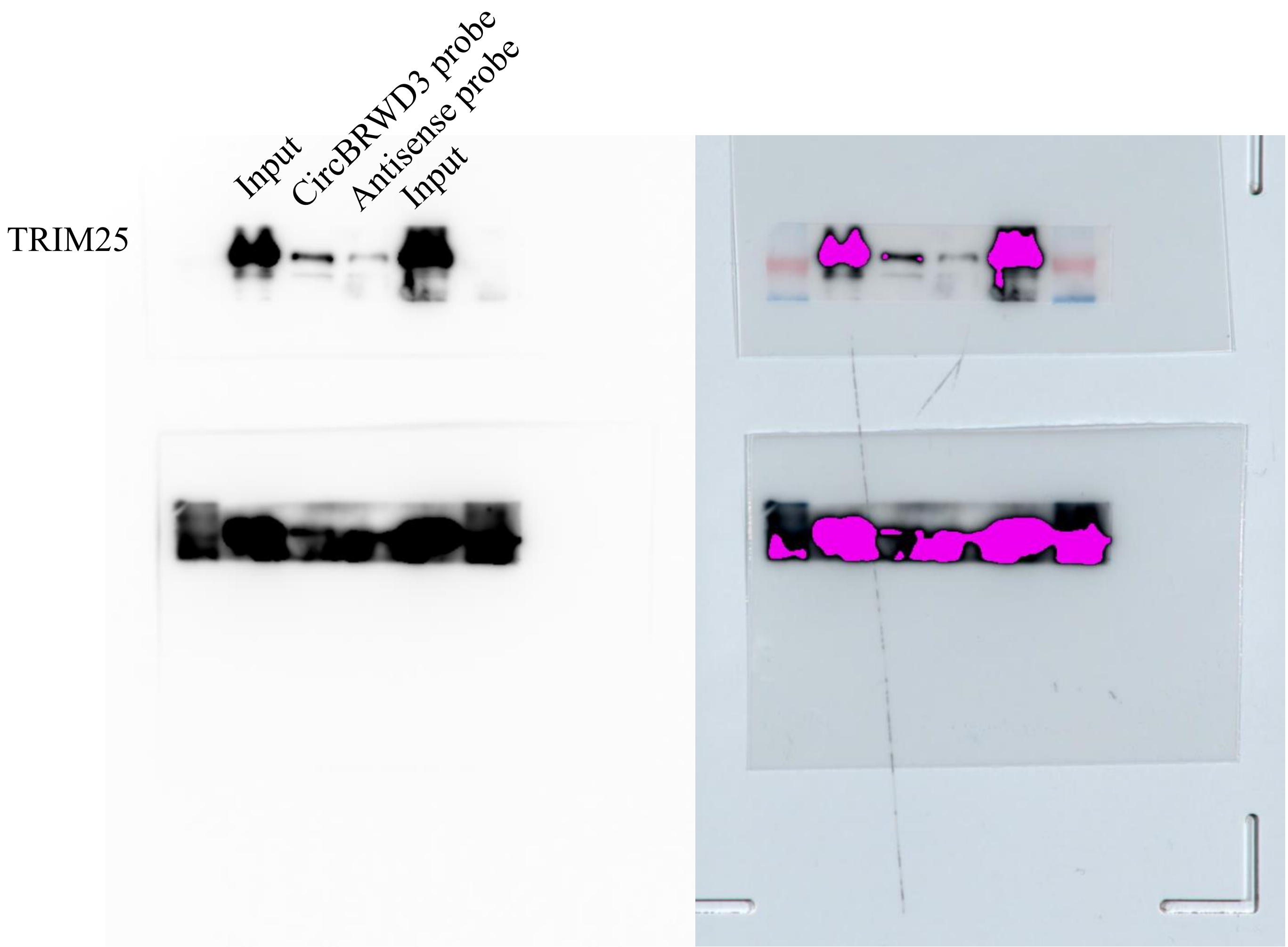

Original Images for Figure 5B

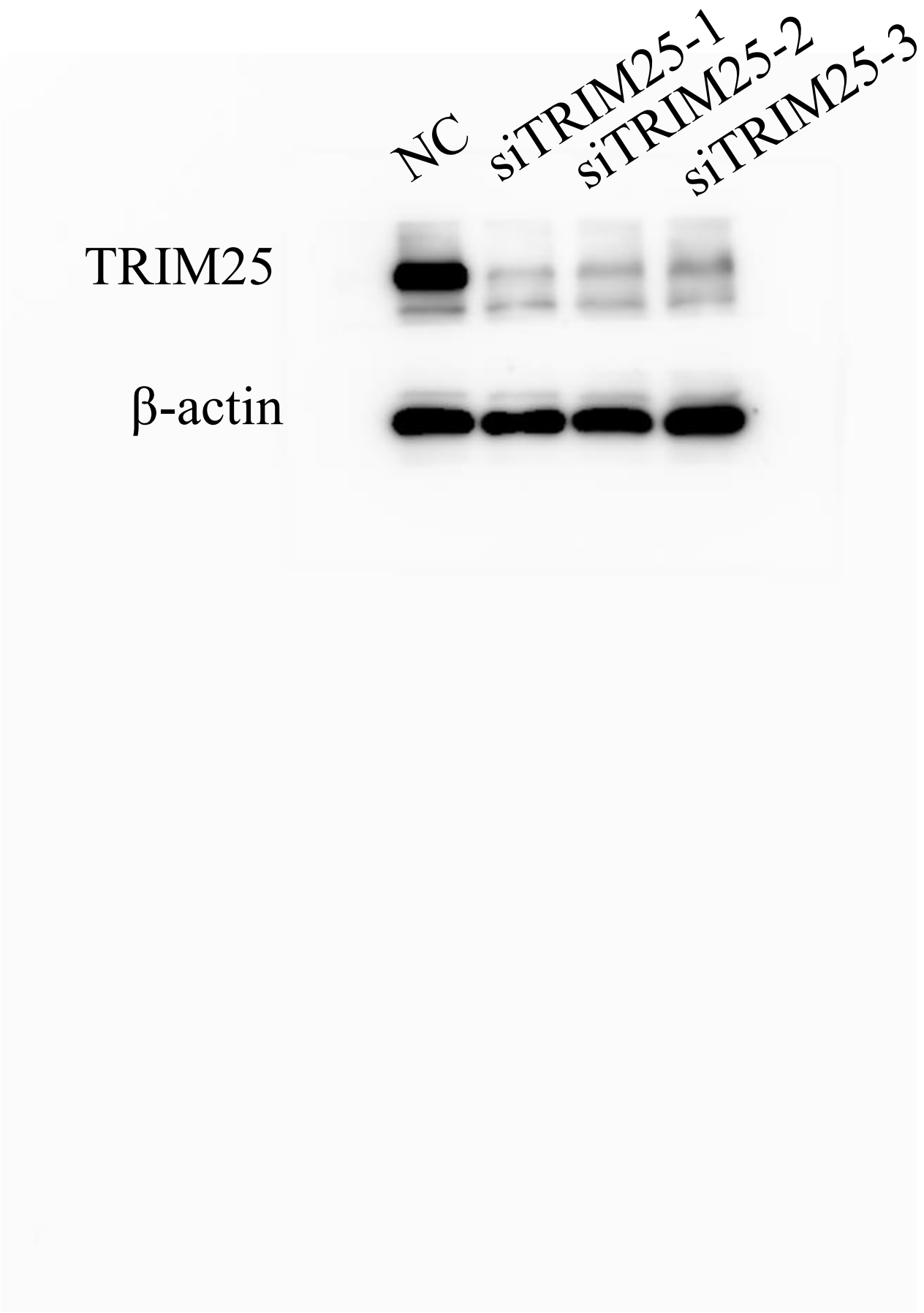

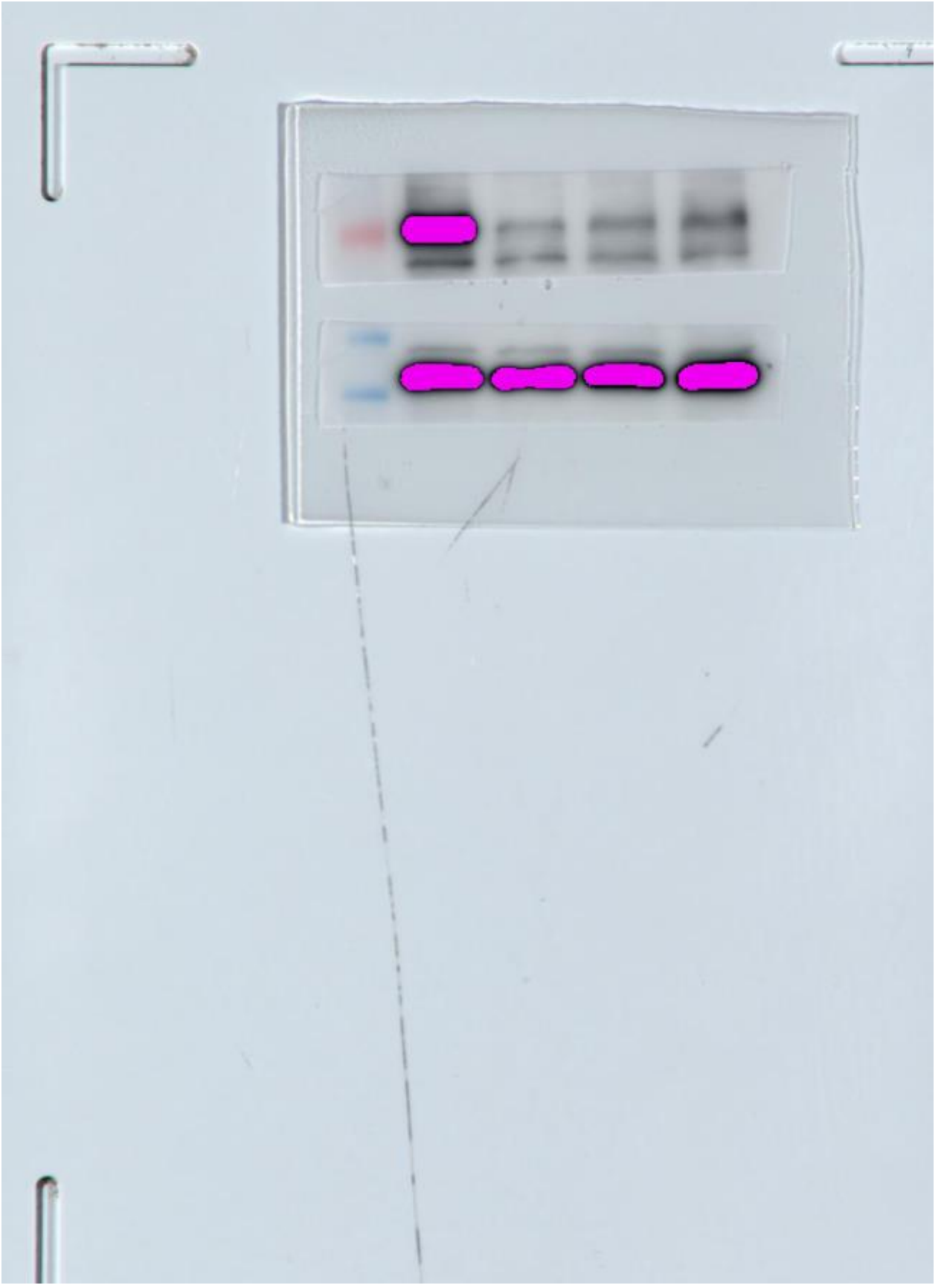

Original Images for Figure 5D

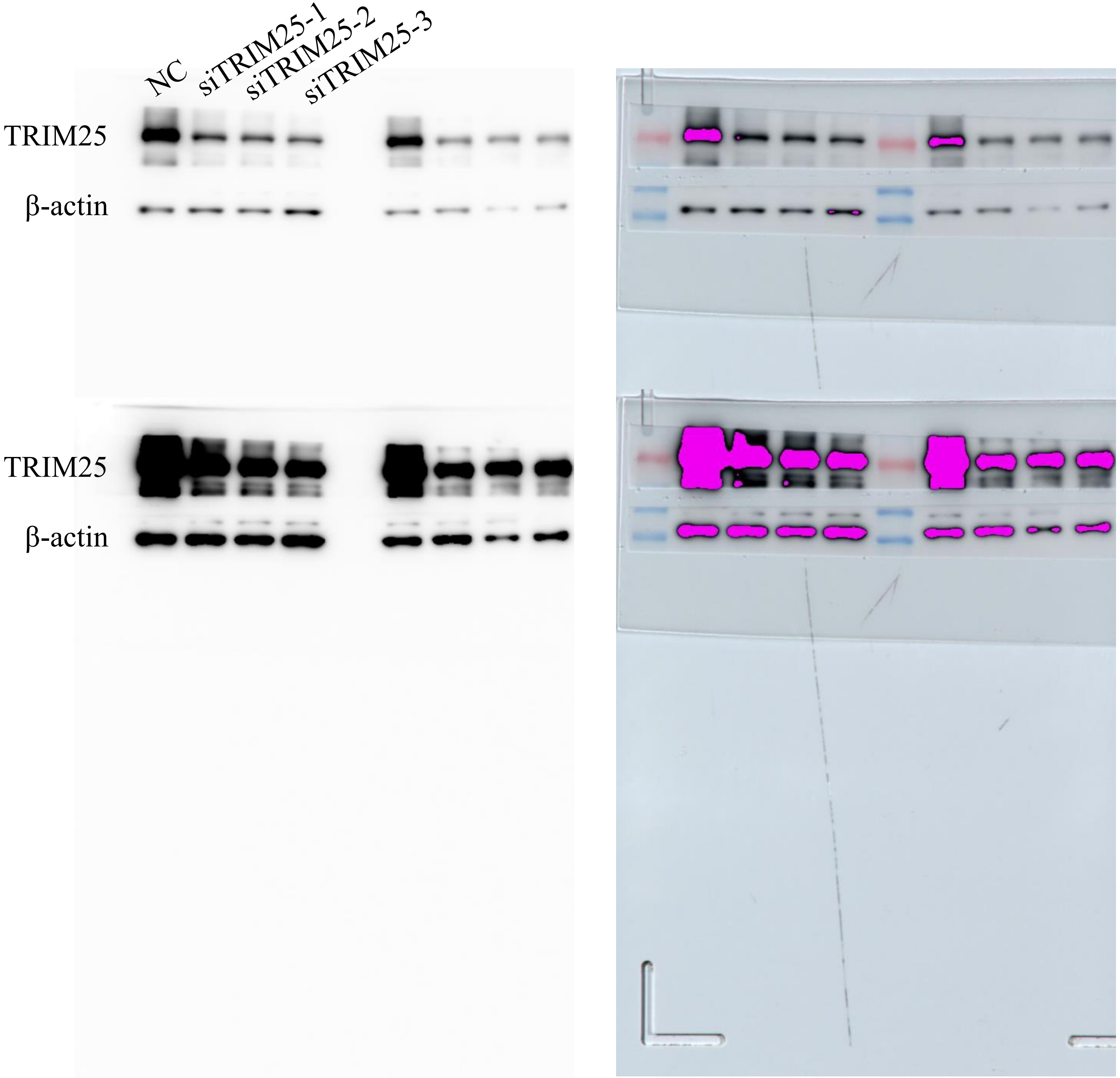

Original Images for Figure 5R

ubiquitin

p53

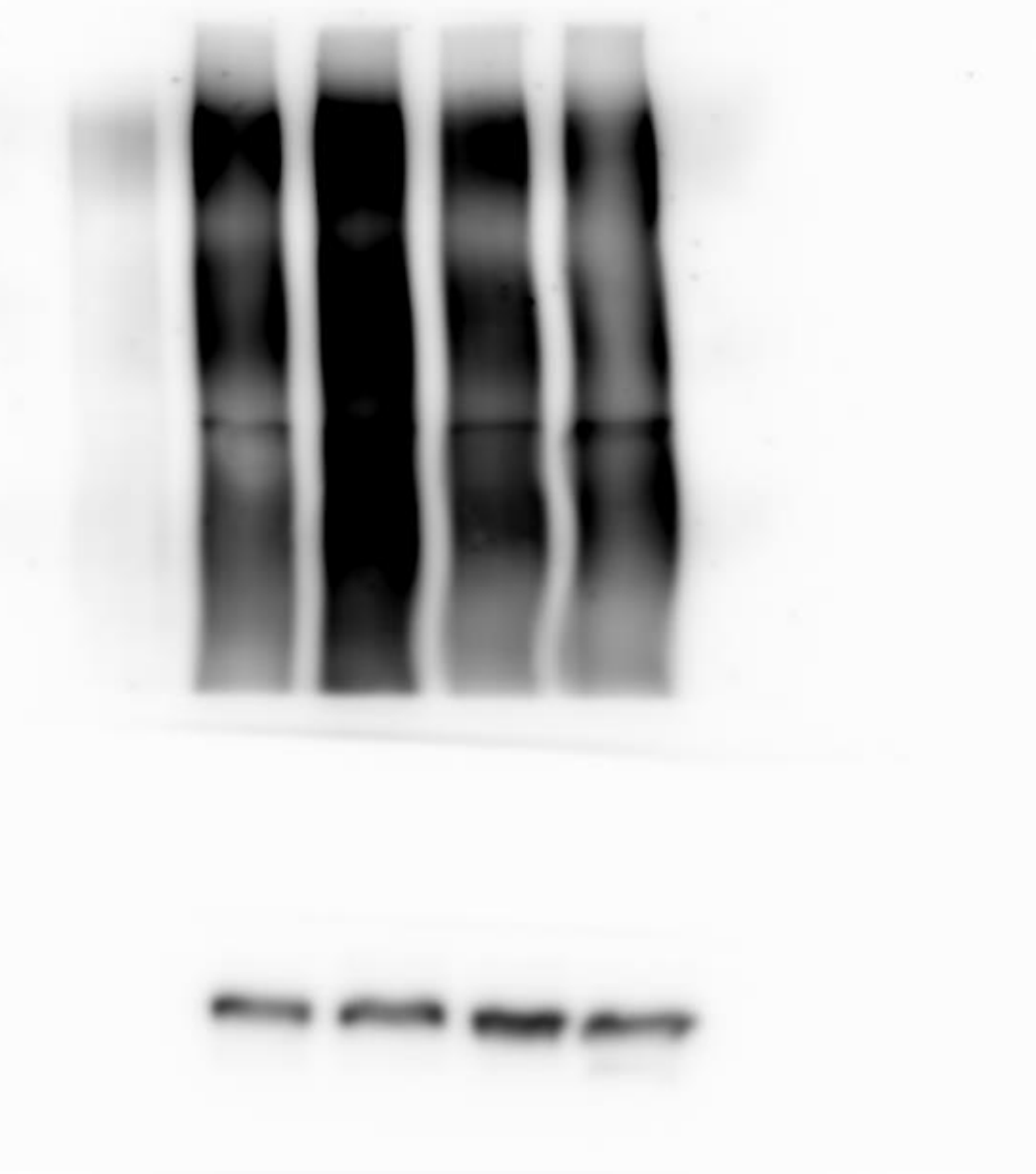

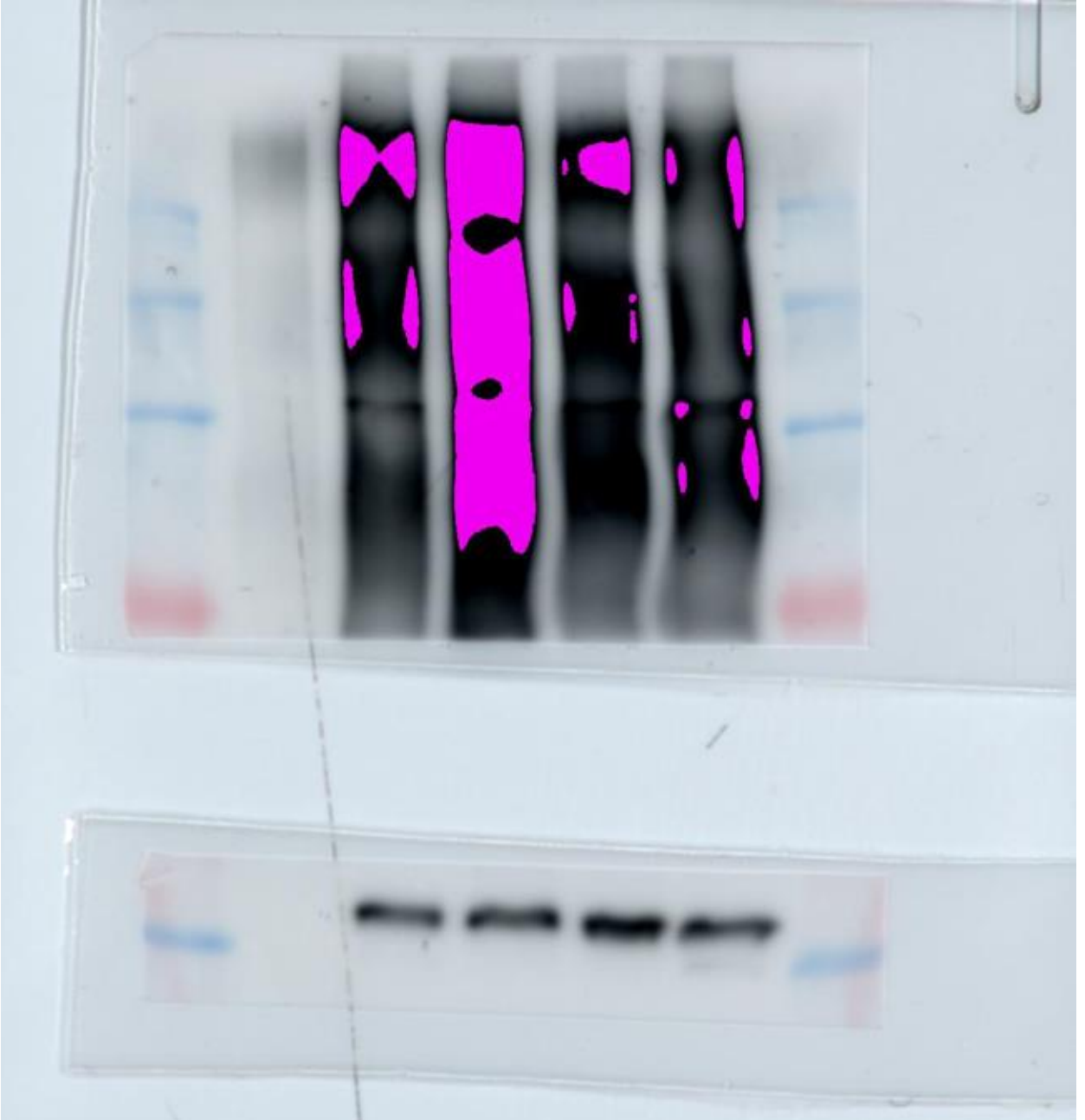

ubiquitin

p53

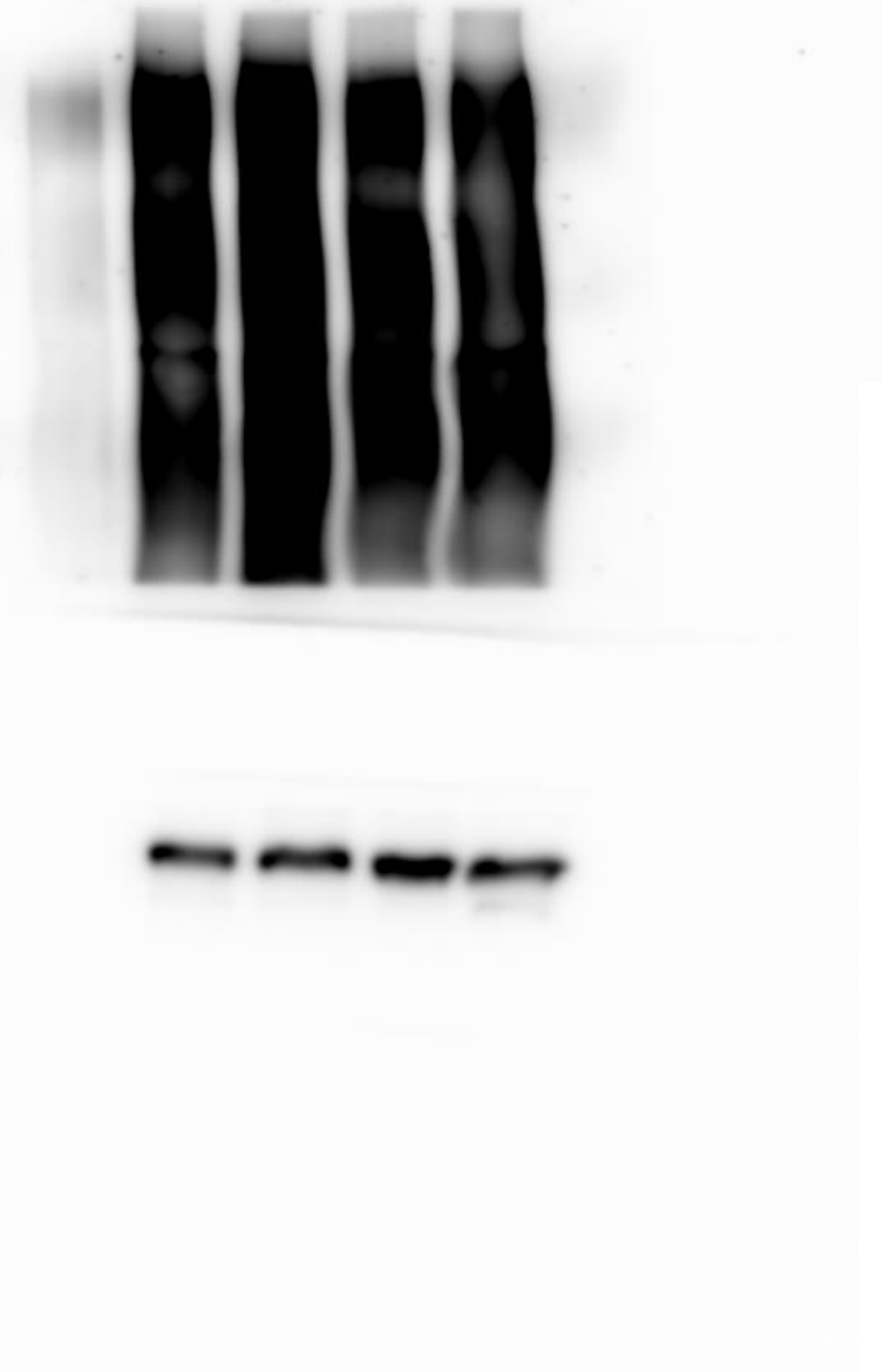

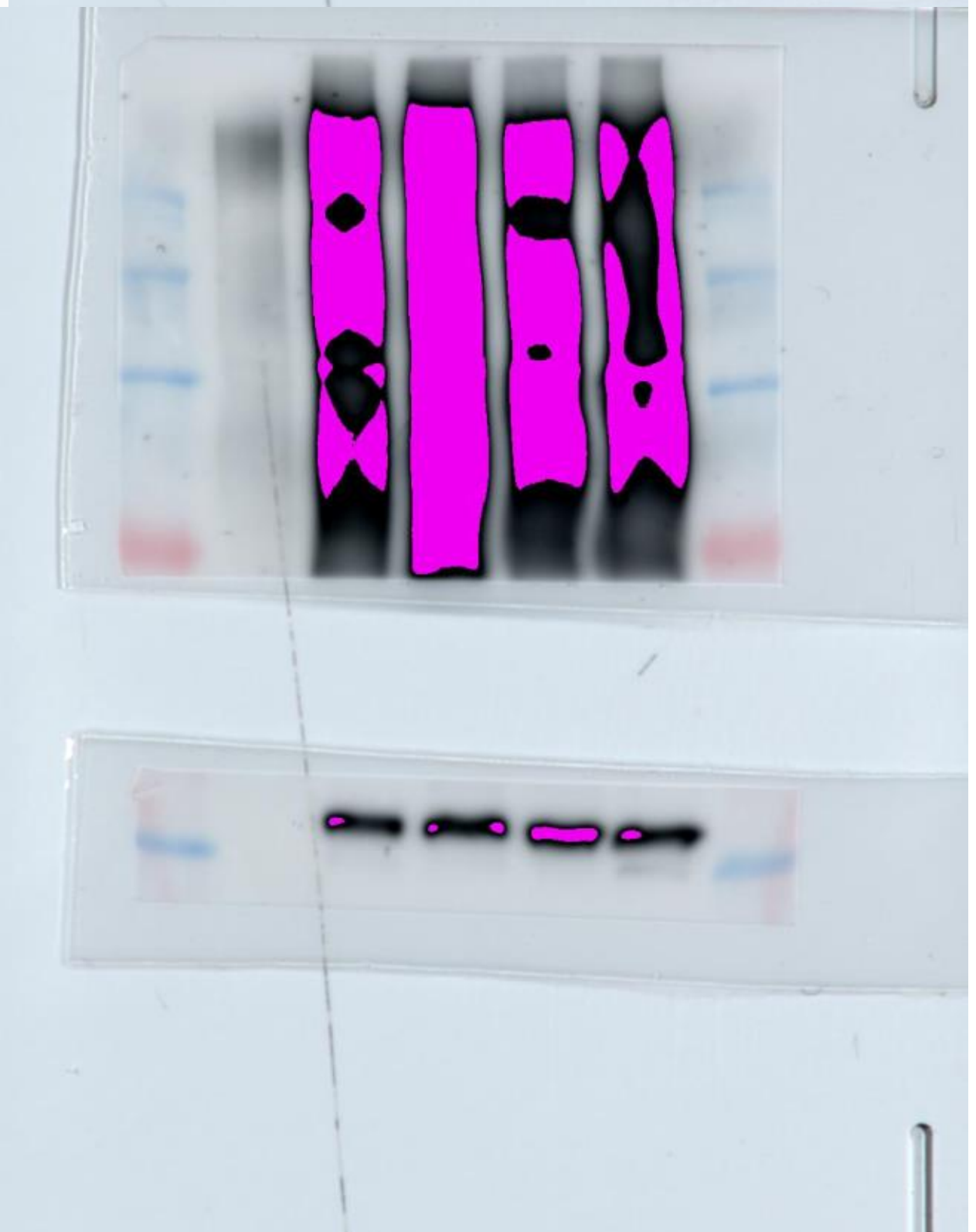

Original Images for Figure 5R

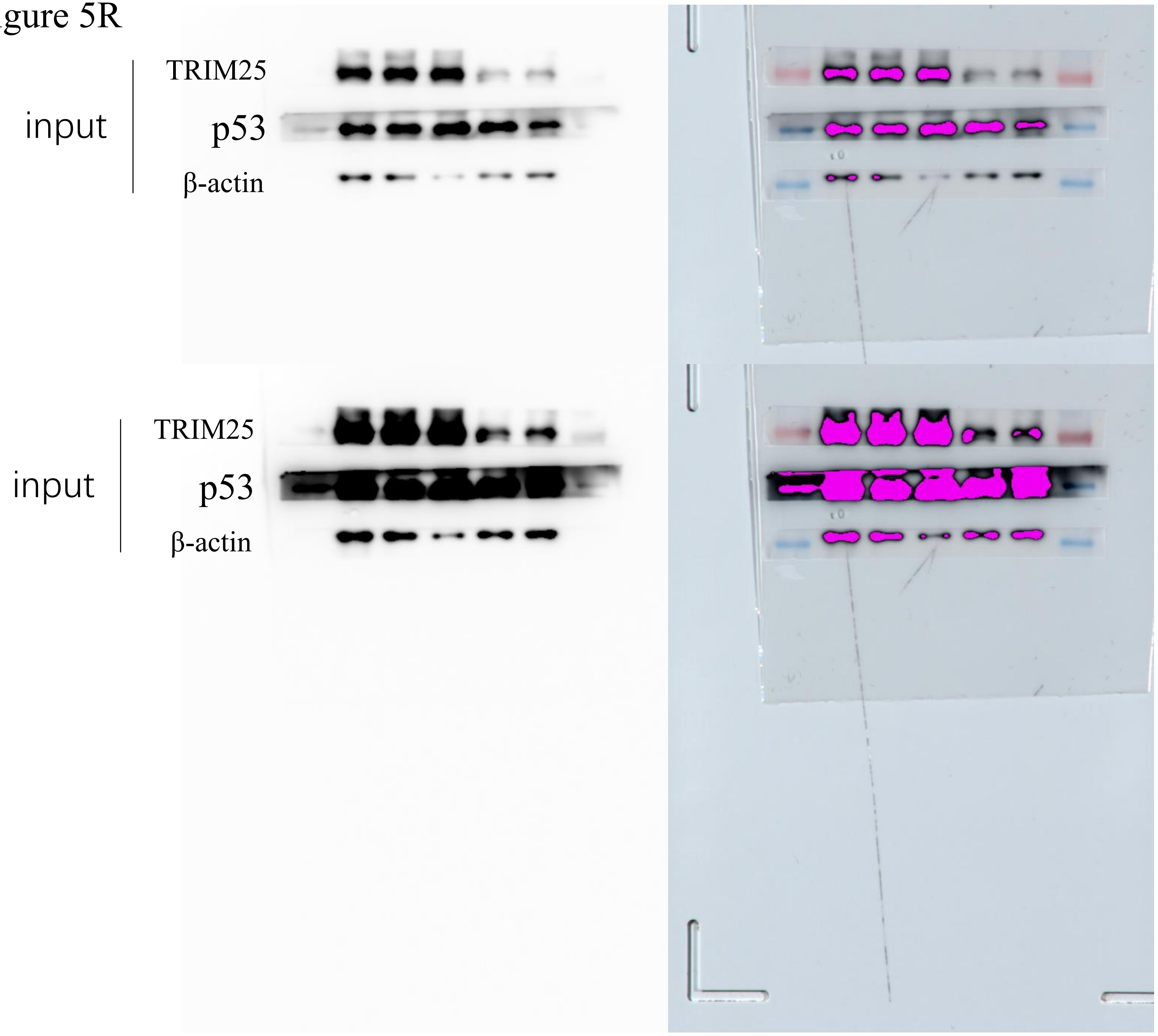

Original Images for Figure 5S

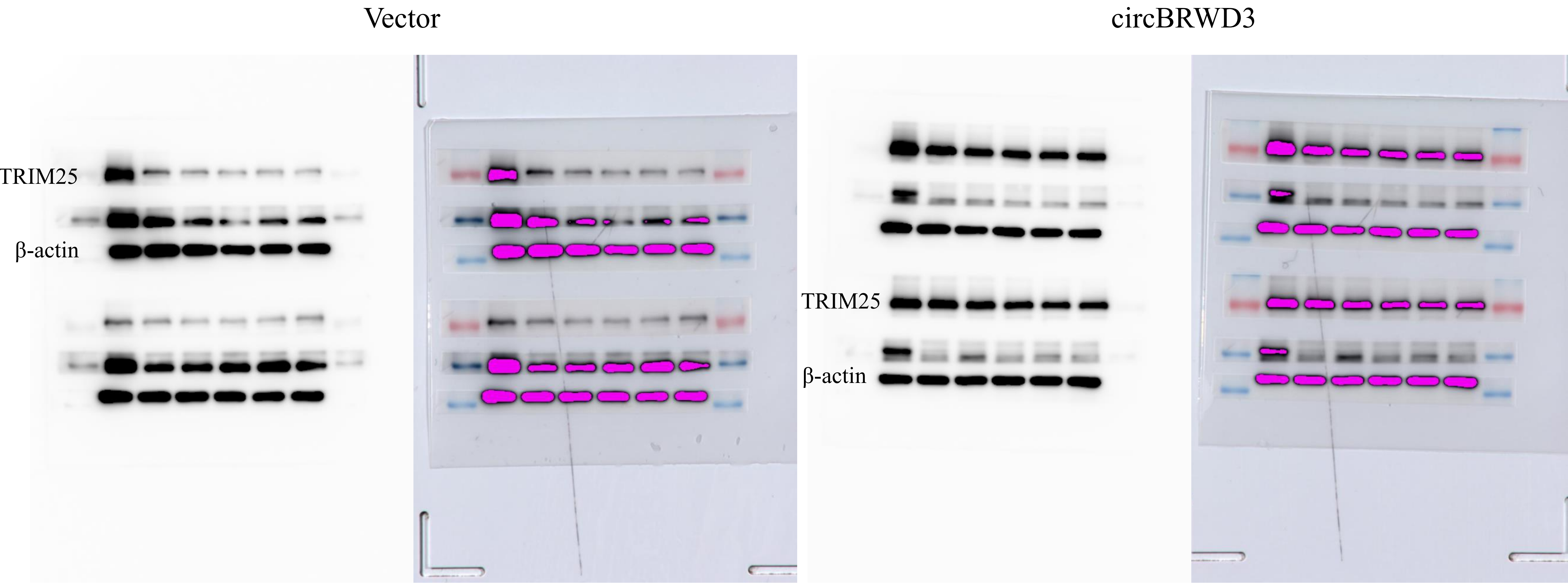

Original Images for Figure 8F

H1299

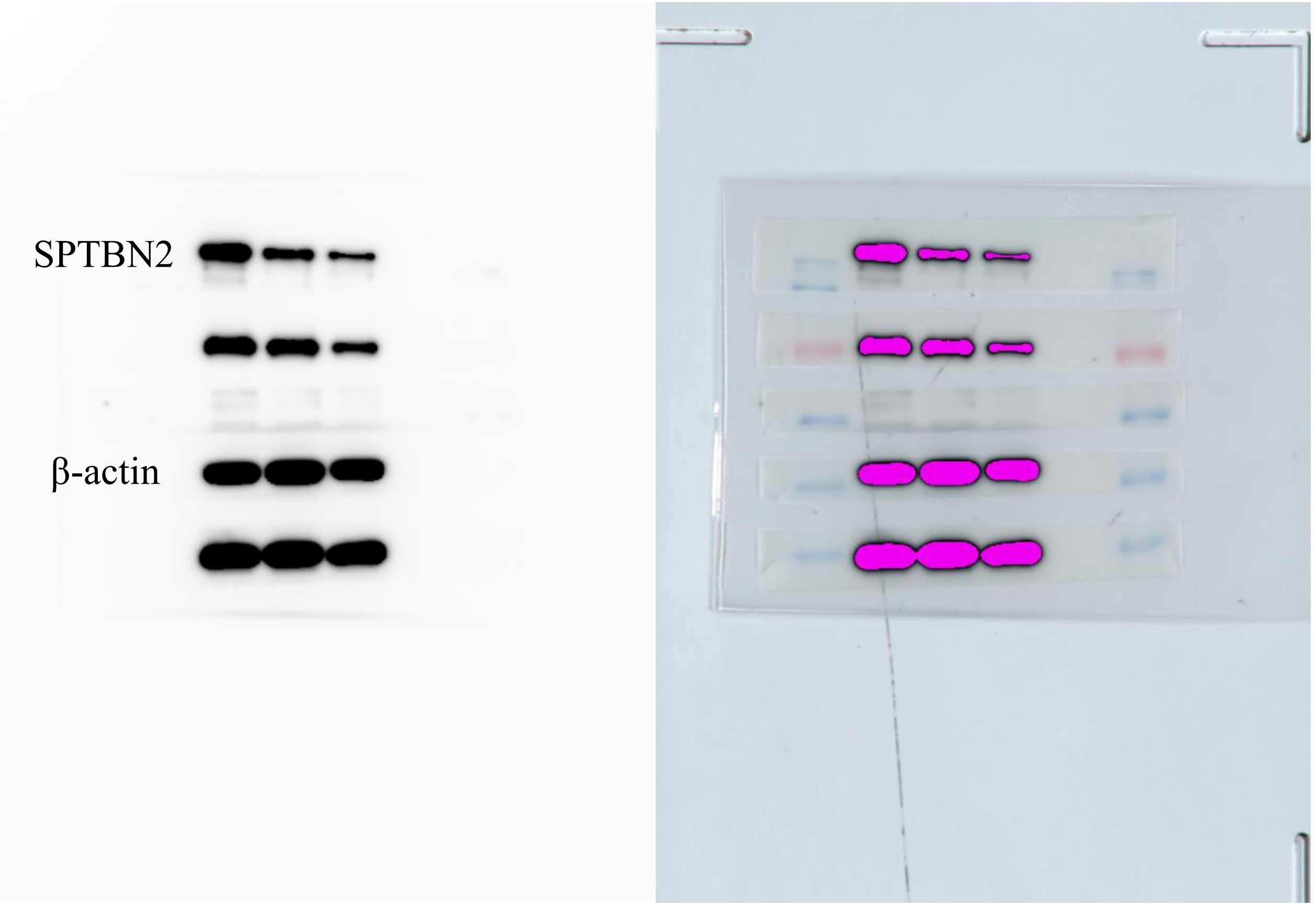

SPC-A1

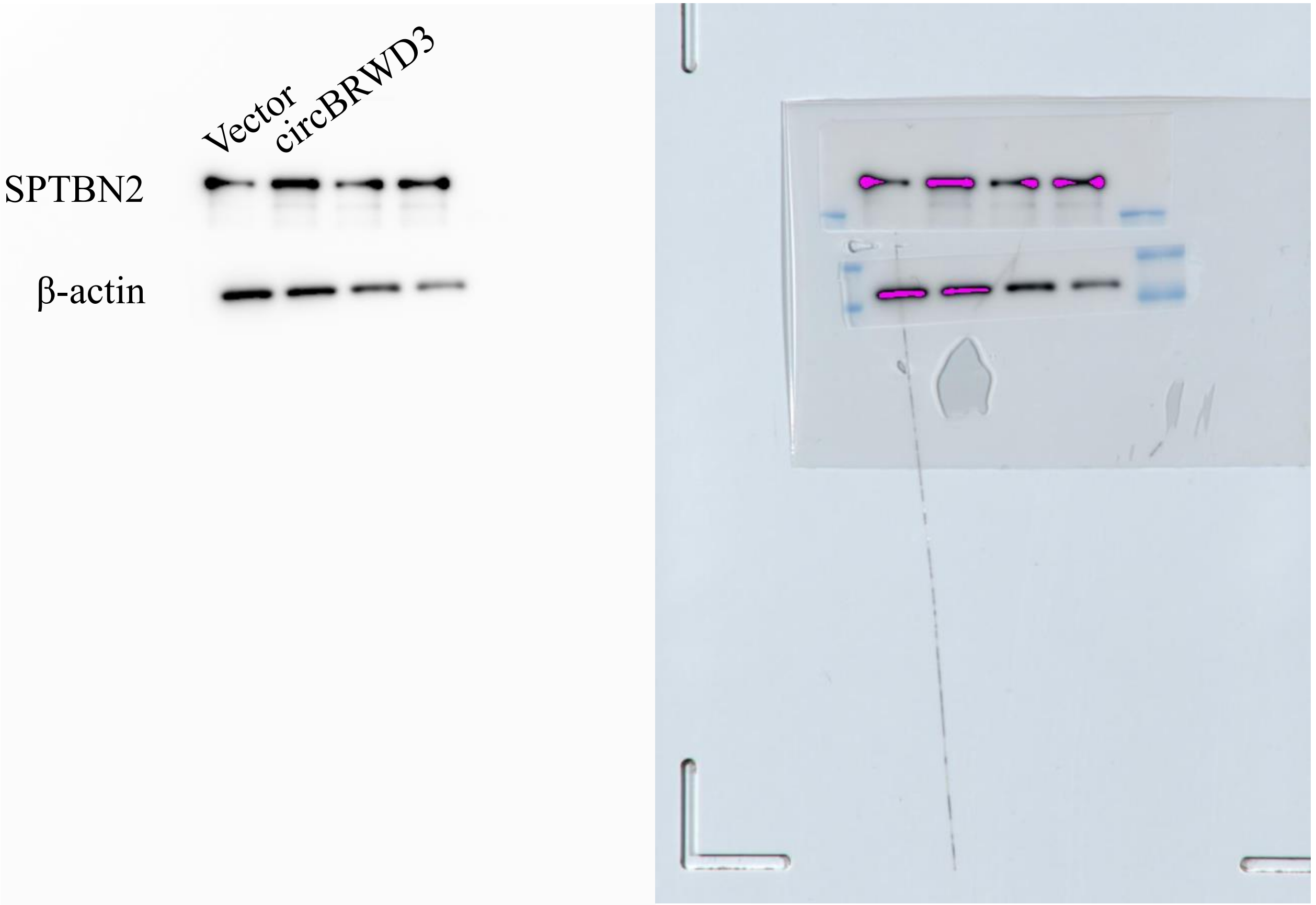

Original Images for Figure 8G

H1299

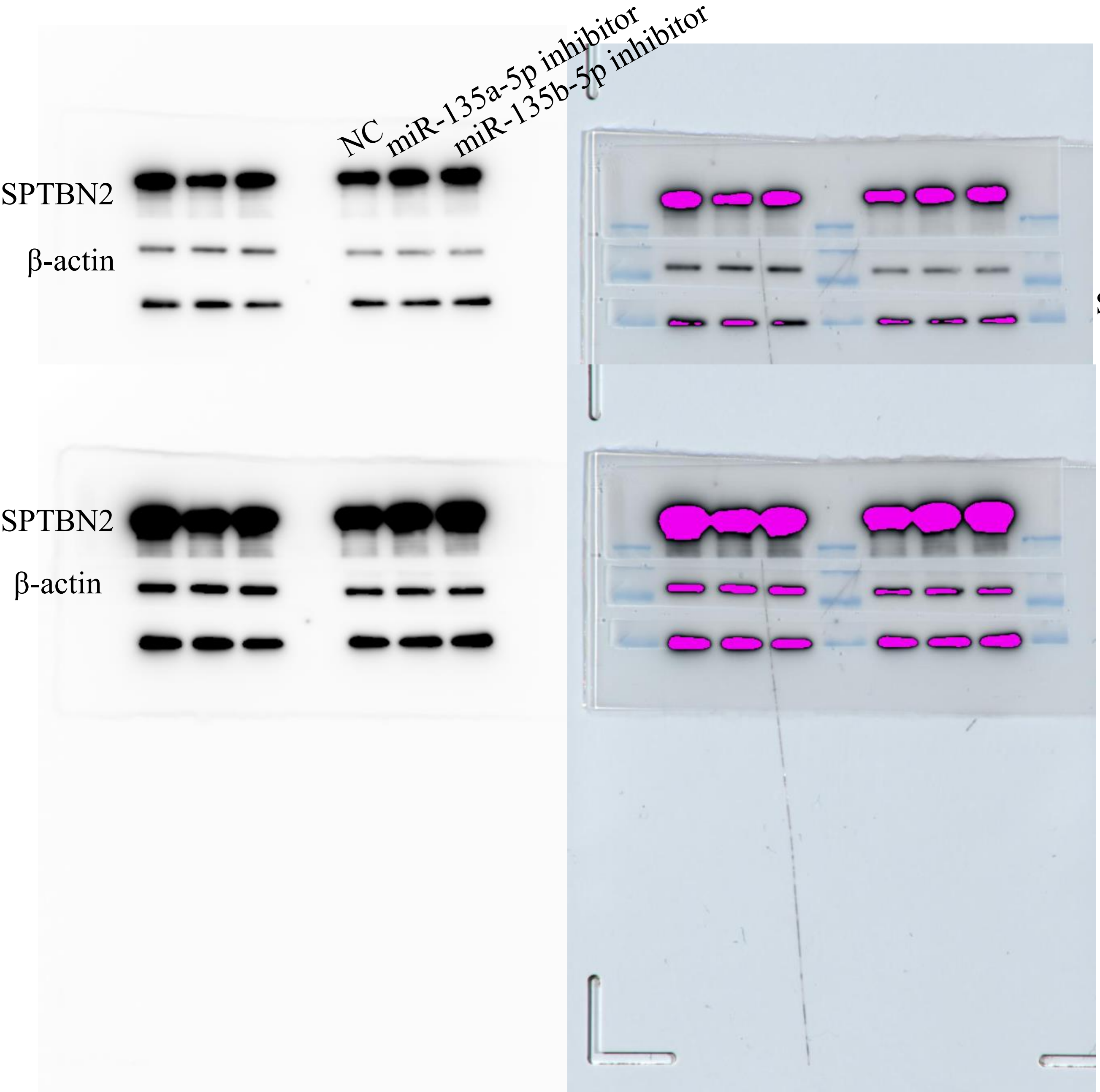

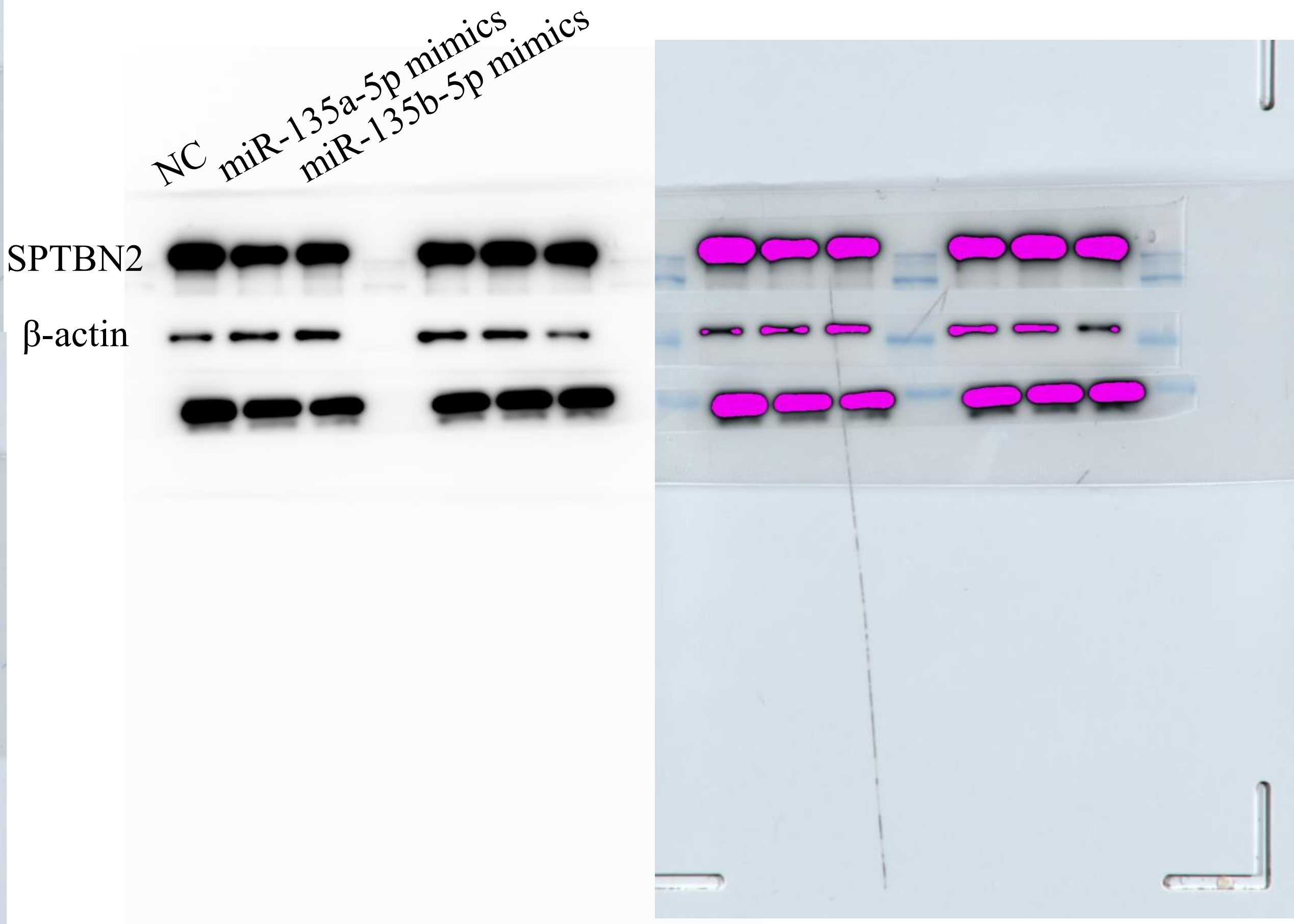

Original Images for Figure 8G

SPC-A1

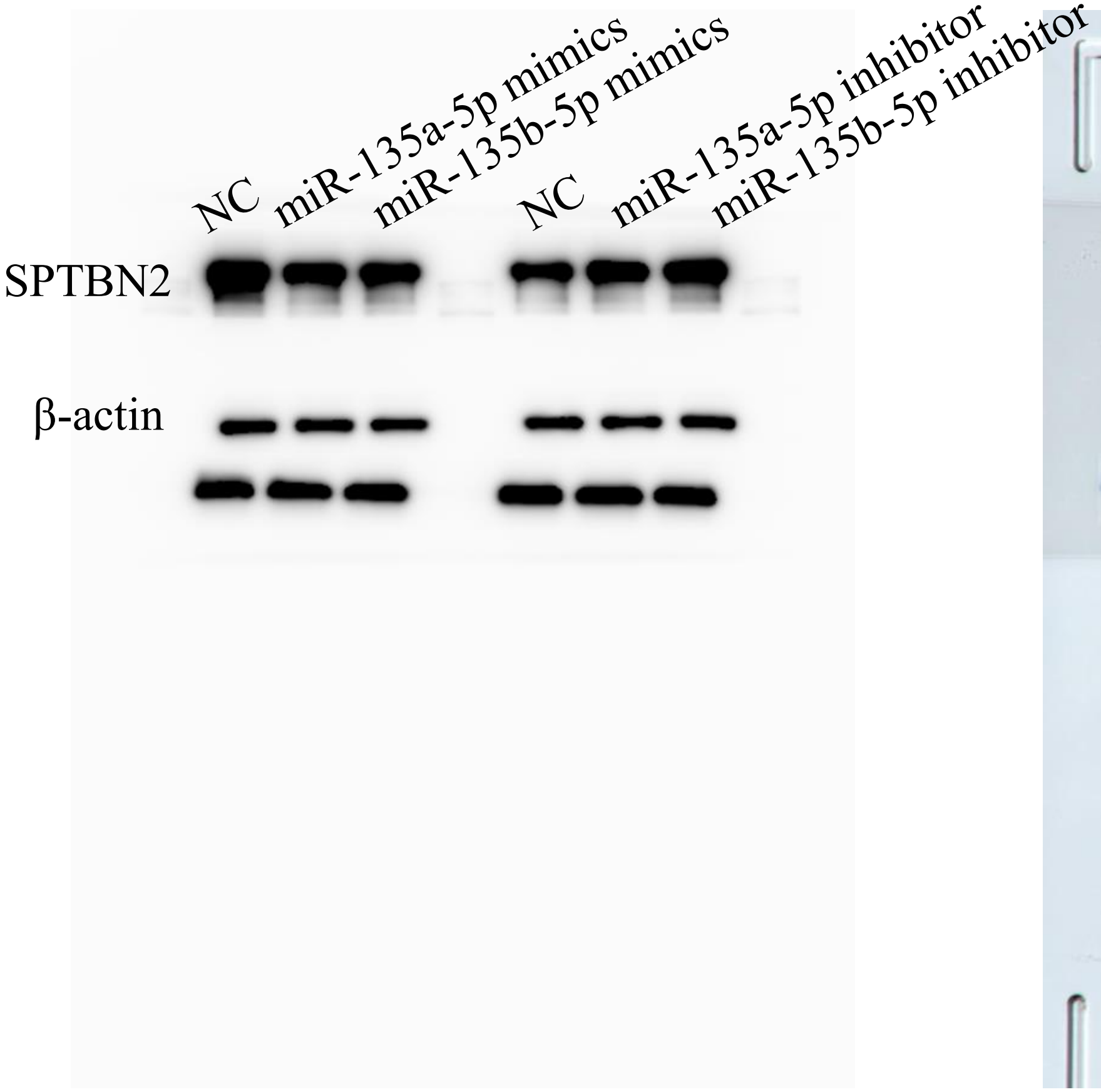

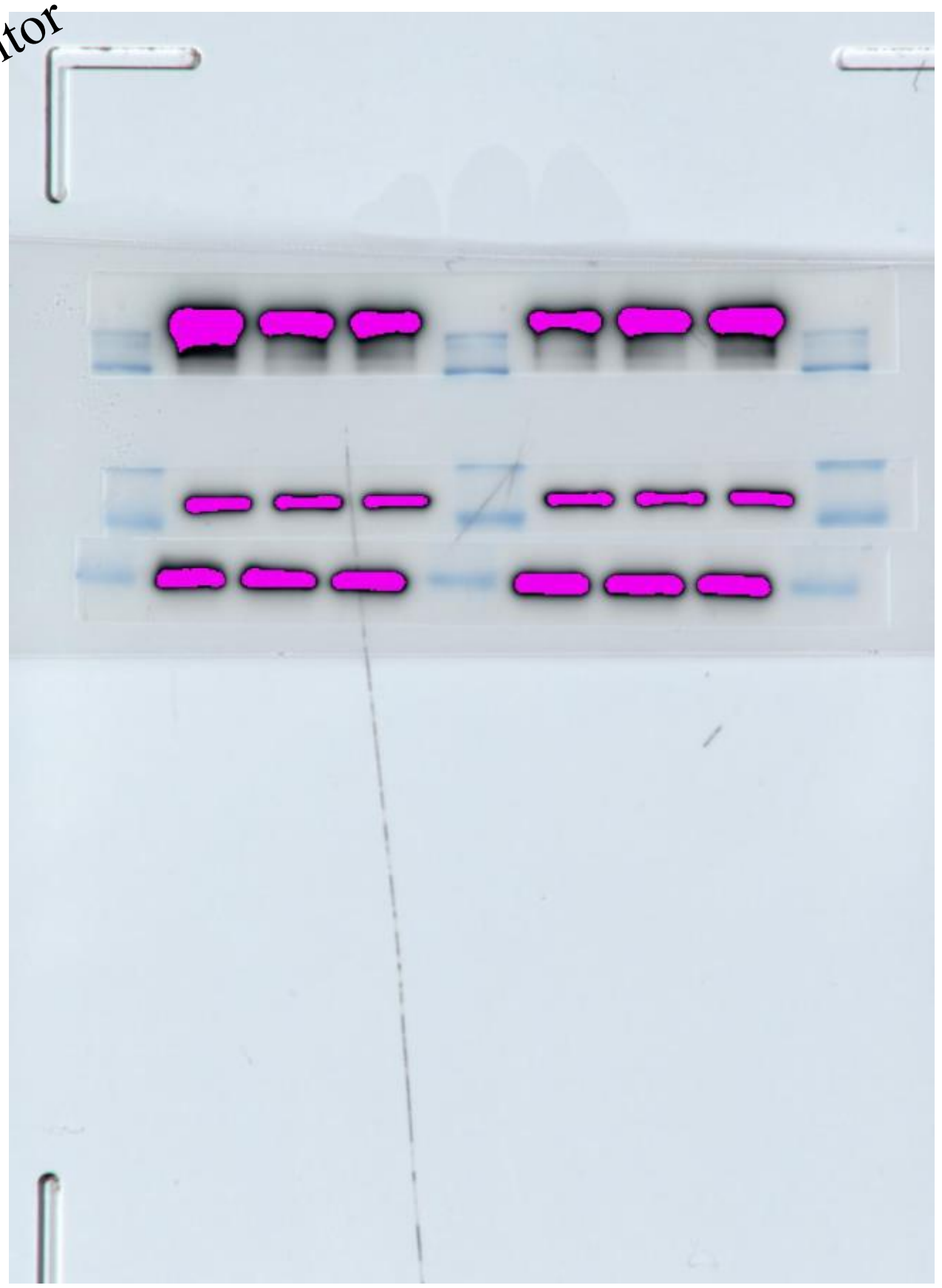

Original Images for Figure 8H

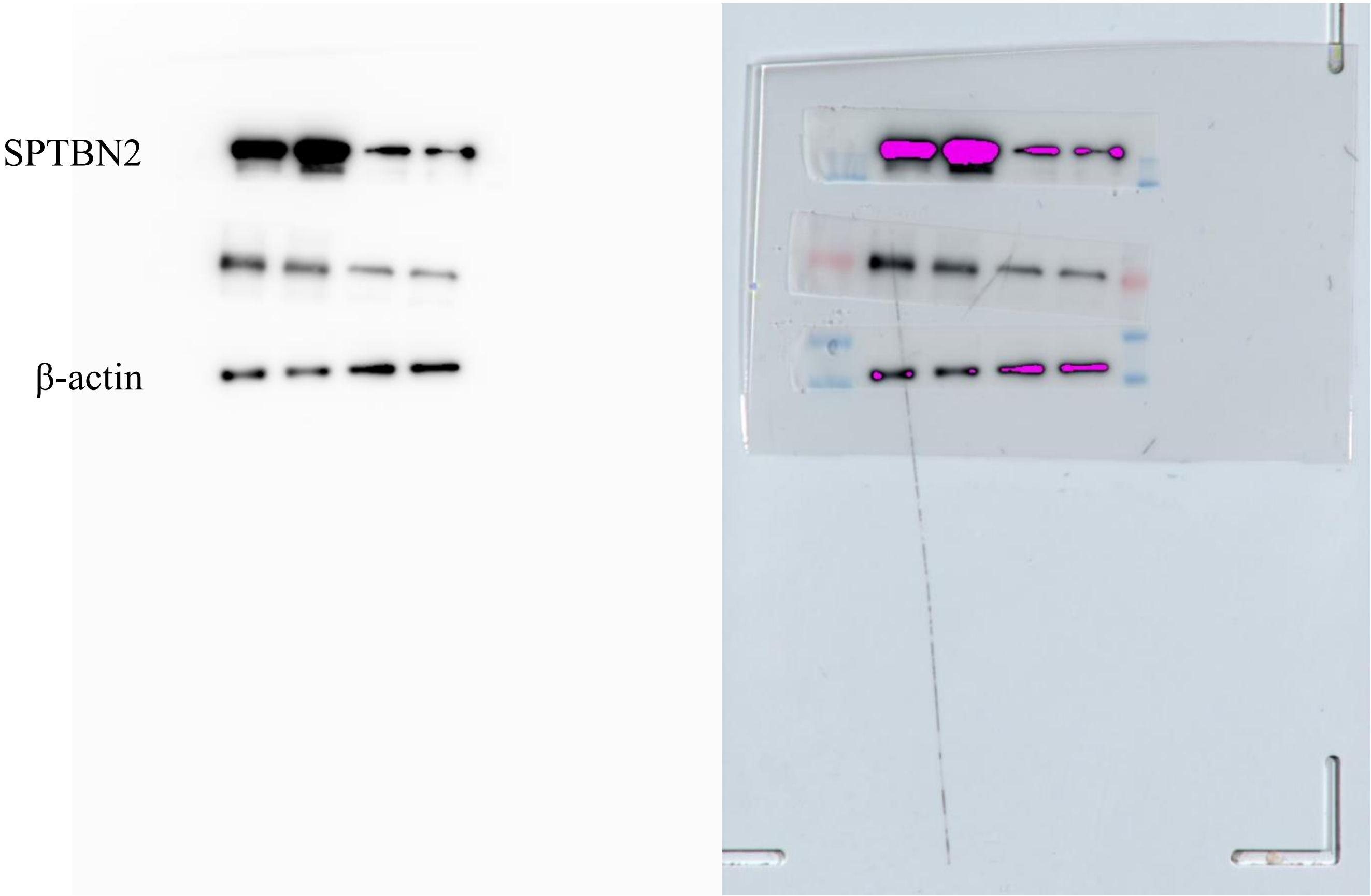

Original Images for Figure 8L

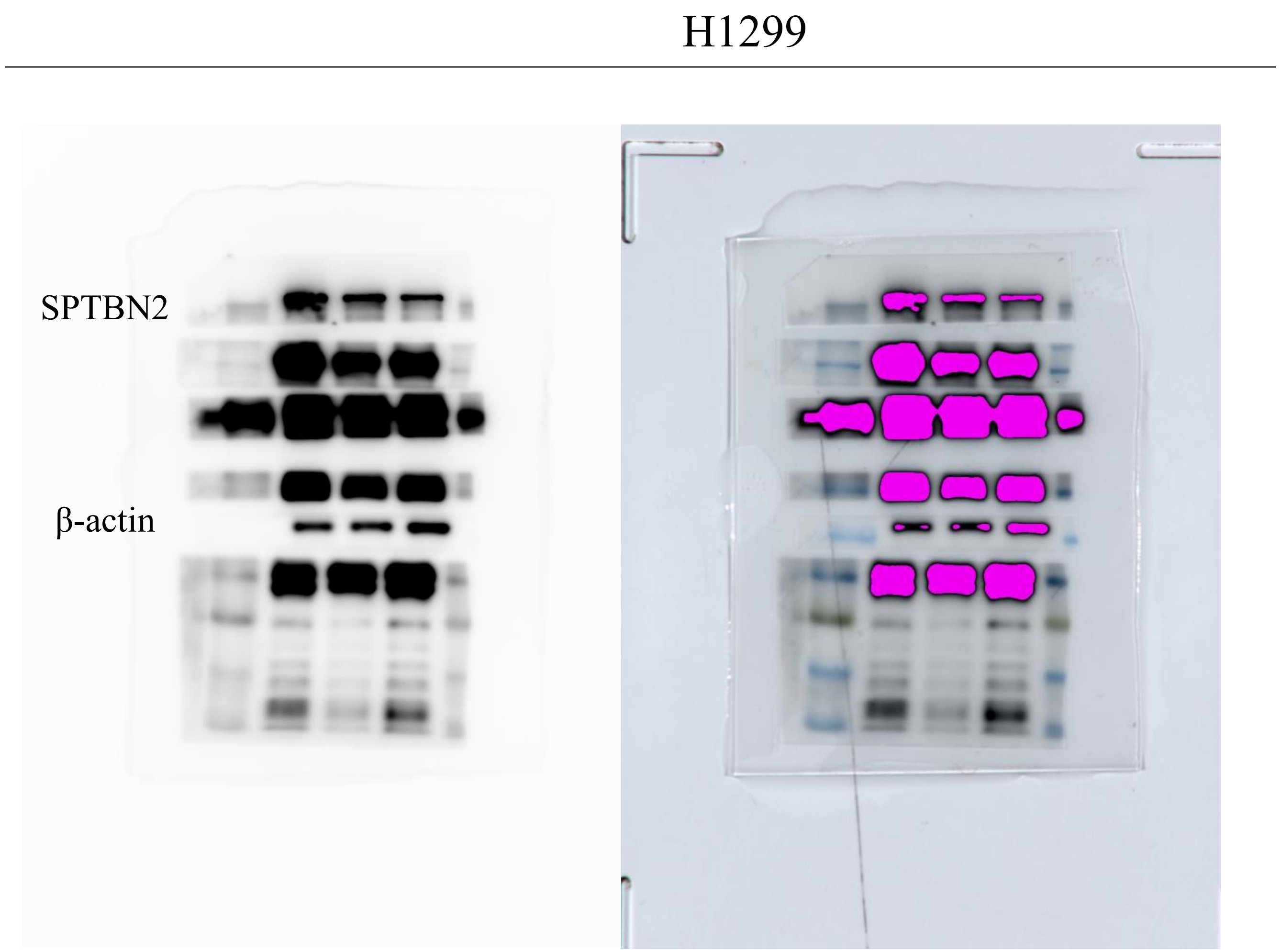

Original Images for Figure 8L

SPC-A1

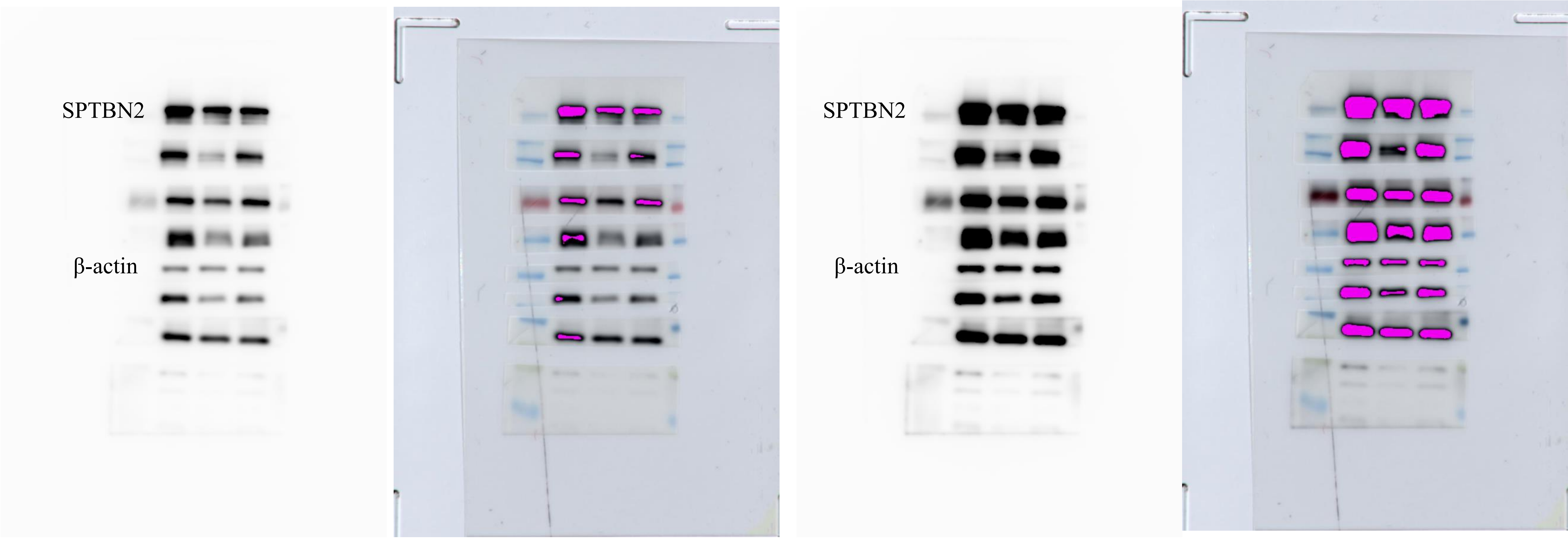

Supplement: Supplementary file 1 [file 41419_2026_9103_MOESM1_ESM.pdf]
